# HIV phylogenetic clusters point to unmet hiv prevention, testing and treatment needs among men who have sex with men in kenya

**DOI:** 10.1186/s12879-024-10052-5

**Published:** 2024-11-20

**Authors:** François Cholette, Lisa Lazarus, Pascal Macharia, Jeffrey Walimbwa, Samuel Kuria, Parinita Bhattacharjee, Helgar Musyoki, Mary Mugambi, Martin K. Ongaro, Kennedy Olango, Janet Musimbi, Faran Emmanuel, Shajy Isac, Michael Pickles, Marissa L. Becker, Sharmistha Mishra, Lyle R. McKinnon, James Blanchard, John Ho, Omari Henry, Rissa Fabia, Paul Sandstrom, Robert Lorway, Souradet Y. Shaw

**Affiliations:** 1 National Sexually Transmitted and Blood Borne Infection Laboratory, National Microbiology Laboratory at J.C. Wilt Infectious Diseases Research Centre, Public Health Agency of Canada, Winnipeg, Canada; 2 Department of Medical Microbiology and Infectious Diseases, University of Manitoba, Winnipeg, Canada; 3 Institute for Global Public Health, University of Manitoba, Winnipeg, Canada; 4 Health Options for Young Men On HIV/AIDS and STIs, Nairobi, Kenya; 5 G10 Research Advisory Committee, Nairobi, Kenya; 6 Mamboleo Peer Empowerment Group, Kiambu, Kenya; 7 Partners for Health and Development in Africa, Nairobi, Kenya; 8 National AIDS and STI Control Programme, Ministry of Health, Nairobi, Kenya; 9 HIV and AIDS People’s Alliance of Kenya, Mombasa, Kenya; 10 Men Against AIDS Youth Group, Kisumu, Kenya; 11 India Health Action Trust, New Delhi, India; 12 Medical Research Council Centre for Global Infectious Disease Analysis, School of Public Health, Imperial College London, London, UK; 13 MAP Centre for Urban Health Solutions, St. Michael’s Hospital, Toronto, Canada; 14 Department of Medicine, University of Toronto, Toronto, Canada; 15 Institute of Medical Sciences, University of Toronto, Toronto, Canada; 16 Institute of Health Policy, Management and Evaluation, University of Toronto, Toronto, Canada; 17 Centre for the AIDS Programme of Research in South Africa, Durban, South Africa; 18 Department of Medical Microbiology, University of Nairobi, Nairobi, Kenya

**Keywords:** Sequence analysis, HIV, Molecular epidemiology, Phylogenetic clustering, Sexual and gender minorities, Kenya

## Abstract

**Background:**

The HIV epidemic in Kenya remains a significant public health concern, particularly among gay, bisexual, and other men who have sex with men (GBMSM), who continue to bear a disproportionate burden of the epidemic. This study’s objective is to describe HIV phylogenetic clusters among different subgroups of Kenyan GBMSM, including those who use physical hotspots, virtual spaces, or a combination of both to find male sexual partners.

**Methods:**

Dried blood spots (DBS) were collected from GBMSM in Kisumu, Mombasa, and Kiambu counties, Kenya, in 2019 (baseline) and 2020 (endline). HIV *pol* sequencing was attempted on all seropositive DBS. HIV phylogenetic clusters were inferred using a patristic distance cutoff of ≤ 0.02 nucleotide substitutions per site. We used descriptive statistics to analyze sociodemographic characteristics and risk behaviors stratified by clustering status.

**Results:**

Of the 2,450 participants (baseline and endline), 453 (18.5%) were living with HIV. Only a small proportion of seropositive DBS specimens were successfully sequenced (*n* = 36/453; 7.9%), likely due to most study participants being virally suppressed (87.4%). Among these sequences, 13 (36.1%) formed eight distinct clusters comprised of seven dyads and one triad. The clusters mainly consisted of GBMSM seeking partners online (*n* = 10/13; 76.9%) and who tested less frequently than recommended by Kenyan guidelines (*n* = 11/13; 84.6%).

**Conclusions:**

Our study identified HIV phylogenetic clusters among Kenyan GBMSM who predominantly seek sexual partners online and test infrequently. These findings highlight potential unmet HIV prevention, testing, and treatment needs within this population. Furthermore, these results underscore the importance of tailoring HIV programs to address the diverse needs of GBMSM in Kenya across different venues, including both physical hotspots and online platforms, to ensure comprehensive prevention and care strategies.

**Supplementary Information:**

The online version contains supplementary material available at 10.1186/s12879-024-10052-5.

## Background

The HIV epidemic in Kenya continues to be a significant public health concern, with approximately 1.4 million individuals living with HIV as of 2021 [1]. Despite notable progress in HIV prevention and treatment efforts, Kenyan gay, bisexual, and other men who have sex with men (GBMSM) bear a disproportionate burden of the epidemic [2, 3], due to several structural factors such as stigma, the criminalization of same-sex relationships, a lack of appropriate services, and barriers to accessing services when they are available [4–6]. By understanding HIV transmission dynamics and pinpointing hotspots of active transmission, as exemplified by HIV phylogenetics [7–9], we can identify areas where prevention, treatment, and testing needs are not being met. Addressing these gaps can facilitate better linkages to HIV prevention and treatment programs, thereby improving health outcomes for GBMSM overall.

In Kenya, HIV prevention efforts have mainly focused on reaching GBMSM in physical spaces (e.g., bars, clubs, and sex dens) [10, 11]. These programs have employed targeted outreach, peer education, as well as condom and lubricant distribution to raise awareness about HIV and provide essential prevention resources to GBMSM [10]. While these initiatives have demonstrated success in reaching GBMSM at physical hotspots, there is an important need to extend these prevention efforts to virtual spaces as well, as seeking sexual partners online is becoming more commonplace [12].

GBMSM who seek partners online pose unique challenges in terms of HIV prevention outreach. They are considered harder to reach compared to GBMSM at physical hotspots due to the anonymity and diversity offered by online platforms [12, 13]. Furthermore, studies have indicated that GBMSM who seek partners online may engage in sexual behaviors with increased risk of HIV transmission compared to their counterparts who exclusively seek partners at physical hotspots. These behaviors may include a higher frequency of condomless anal sex with male partners [14–16], a greater number of sexual partners [13, 14], and substance use [17]. In other cases, a significant proportion of GBMSM who seek partners online (> 70%) are unaware of their HIV status, despite being well connected to GBMSM-focused community-based organizations [13, 18]. Despite the lack of a definitive profile for GBMSM who use virtual platforms to find sexual partners, they appear to be younger, unemployed, and have higher educational attainment [13, 14, 19]. Some of our earlier work suggests that HIV prevalence among GBMSM who seek partners online may be much higher compared to GBMSM who solely seek partners at physical hotspots (e.g., 26.7% versus 8.5%) [13]. This higher prevalence of HIV within the subgroup of GBMSM who prefer online hotspots underscores the need to address the specific transmission dynamics within this population.

This study’s objective is to describe HIV phylogenetic clusters among different subgroups of Kenyan GBMSM who use physical hotspots, online platforms, or a combination of both to find sexual partners. The HIV sequences in our dataset are part of a previous study in Kenya investigating the effectiveness of a community-led HIV self-testing delivery strategy among GBMSM to reduce the number of undiagnosed HIV cases and enhance linkage to prevention and treatment services [6].

## Methods

### Study participants

GBMSM were recruited from physical and virtual hotspots in Kisumu (*n* = 811), Mombasa (*n* = 818), and Kiambu (*n* = 821) counties, Kenya during April to June 2019 (baseline) and July to September 2020 (endline) using a randomized multistage sampling approach [6]. The study was embedded within existing community-based HIV prevention and treatment programs for GBMSM. The inclusion criteria were: identifying as male, engaging in anal or oral sexual intercourse with another male in the previous 12 months, and being aged 15 years or older. Lists of recruitment hotspots were developed with the assistance of community-based organizations and GBMSM staff affiliated with those organizations. All consenting participants completed face-to-face questionnaires (Supplementary File 1), underwent HIV rapid testing (Alere Determine HIV-1/2 Combo, Murex HIV1-2-O First Response Kit) and counselling by an HIV testing service counselor as per Kenya national guidelines [6]. If their HIV test was positive, participants were asked to provide a dried blood spot (DBS) specimen from finger pokes for HIV confirmatory serology (HIV National Laboratory, Nairobi, Kenya). DBS were initially screened using the Bioelisa HIV-1 + 2 Ag/Ab (Werfen, Barcelona, Spain) test, followed by confirmatory testing using the Murex HIV1-2-O (DiaSorin, Saluggia, Italy) test. In cases of discordant results between the screening and confirmatory tests, both tests were repeated to serve as a tie-breaker. HIV positive DBS specimens were shipped on dry ice to the National Sexually Transmitted and Blood-Borne Infections Laboratory (Public Health Agency of Canada, Winnipeg, Canada) for subsequent HIV RNA quantification (Aptima HIV-1 Quant Dx Assay; Hologic, Mississauga, Canada) and HIV *pol* sequencing.

### HIV pol sequencing

A portion of the HIV *pol* gene (position 2147 to 3326 on HXB2 accession no. K03455) was sequenced using a routine, in-house, HIV drug resistance mutation genotyping assay for strain and drug resistance determination [20, 21].

Total nucleic acid was isolated from one entire dried blood spot (~ 75 µL) using a NucliSENS EMAG instrument (bioMérieux, Marcy-l’Étoile, France) according to the manufacturer’s protocol for whole blood (no. WF1A). Purified nucleic acid was eluted in 50 µL of NucliSENS Extraction Buffer 3 (bioMérieux) and stored immediately at -80ºC until further processing.

The partial HIV *pol* gene was amplified by reverse transcription polymerase chain reaction (RT-PCR) and nested PCR. RT-PCR was performed on 10 µL of purified nucleic acid using the SuperScript IV One-Step RT-PCR System (ThermoFisher, Ottawa, Canada) in 50 µL reactions containing 25 µL of 2X Platinum SuperFi RT-PCR Master Mix, 1 µL of forward primer (10 µM; 5’-GAR AGA CAG GCT AAT TTT TTA GGG A-3’), 1 µL of reverse primer (10 µM; 5’-ATC CCT GCA TAA ATC TGA CTT GC-3’), 0.5 µL of SuperScript IV RT Mix, and 22.5 µL of nuclease-free water. Thermal cycling conditions were as follows: 50˚C/10 min, 98˚C/2 min, followed by 40 cycles of 98˚C/20 s, 50˚C/30 s, 72˚C/1 min, and a final extension at 72˚C/5 min. Nested PCR was performed on 5 µL of RT-PCR product using the Invitrogen Platinum II Hot-Start PCR Master Mix (ThermoFisher) in 50 µL reactions containing 25 µL of Platinum II Hot-Start PCR Master Mix (2X), 2 µL of forward primer (10 µM; 5’-CAG AGC CAR CAG CCC CAC C-3’), 2 µL of reverse primer (10 µM; 5’-CTT CTG TAT GTC ATT GAC AGT CC-3’), and 16 µL of nuclease-free water. Thermal cycling conditions were as follows: 94˚C/2 min, followed by 40 cycles of 94˚C/15 s, 60˚C/15 s, 68˚C/30 s, and a final extension at 68˚C/5 min. All nested PCR products were quantified using the Qubit 1X dsDNA HS Assay Kit (ThermoFisher) and purified using AMPure XP solid phase reversible immobilization paramagnetic beads (Beckman Coulter, Indianapolis, US) on an epMotion 5075t liquid handling workstation (Eppendorf, Hamburg, Germany) prior to sequencing.

Sequencing libraries were prepared using the Nextera XT DNA Library Preparation Kit (Illumina, San Diego, US). A maximum of 96 libraries were pooled per sequencing run with a 5% spike-in of PhiX Control v3 library (Illumina). All steps of the library preparation were performed using an epMotion 5075t liquid handling workstation (Eppendorf). Sequencing was done on an Illumina MiSeq platform with the MiSeq Reagent Kit v2 (300 cycles; Illumina) according to the manufacturer’s instructions. HyDRA Web (https://hydra.canada.ca), an open-source pipeline for analyzing next generation sequencing (NGS) sequencing data, was used for sequence analysis, quality control, and contig assembly [20]. Each consensus sequence was subtyped using a combination of COMET HIV-1 and maximum likelihood phylogenetic inferences made with HIV subtype reference sequence alignments from the Los Alamos National Laboratory HIV database [22, 23].

### Cluster analysis

Putative HIV transmission clusters were inferred by using patristic distances. Subtype-specific alignments were built in in Geneious Prime (Biomatters Inc., Boston, USA) with MAFFT [24]. In addition to the study sequences, all available Kenyan HIV *pol* sequences from the Los Alamos National Laboratory HIV database corresponding to the respective subtypes were included as background sequences, along with the top 25 BLASTn hits for each study sequence (Supplementary File 2). Duplicate sequences were removed from alignments in Geneious Prime (Biomatters Inc.) using the “Find Duplicates” function and “Sequences with identical residues” option. Maximum likelihood phylogenetic trees were reconstructed with IQ-TREE v1.6.1 [25] using the best-fitting substitution model selected by ModelFinder [26]. Reliability of the tree topologies was inferred by ultrafast boostrap (UFBoot) re-sampling (*n* = 100,000) [27]. We calculated patristic distances directly from the phylogenies in Geneious Prime (Biomatters), defining clusters as sequences with a patristic distance of ≤ 0.02 nucleotide substitutions per site [7, 28]. Given the small sample size in our study, we manually inspected the distance matrices to identify clusters. No automated scripts or tools were used in this step. Distance matrices were also exported as.csv files, then imported, annotated, and visualized in Cytoscape v3.9.1 [29].

### Descriptive analysis

Study participants’ sociodemographic characteristics are reported using descriptive statistics. Continuous data are summarized using the median and interquartile range (IQR). Categorical data are presented using exact numbers and proportions. All analyses were conducted using SPSS Statistics v28 (IBM, Armonk, United States).

## Results

**Fig. 1:**
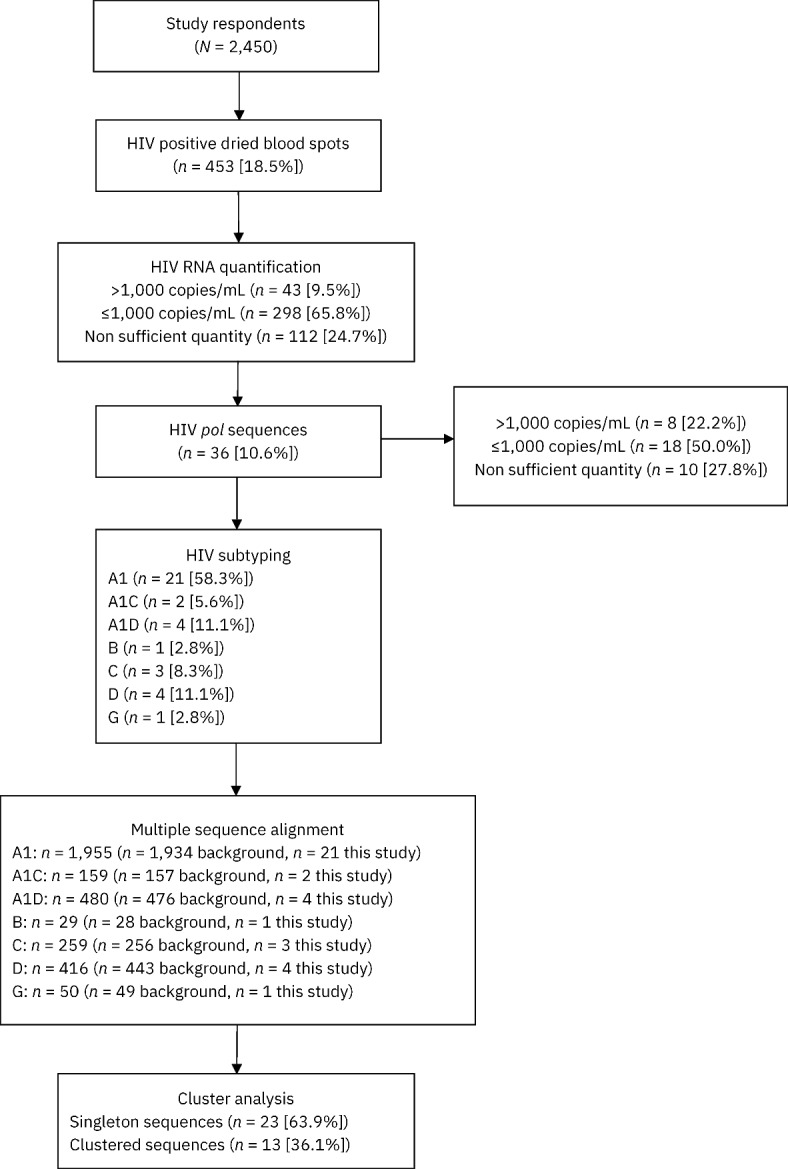
Study workflow. Due to the nature of the study, HIV *pol* sequencing was prioritized over HIV RNA quantification. Consequently, for some of the 36 participants with available HIV *pol* sequences, viral load measurements were missing due to insufficient sample quantity remaining after sequencing

A total of 2,450 participants consented to complete the behavioural survey throughout the entire study period, and all participants who tested positive during rapid HIV testing provided a DBS sample. Serological testing identified *n* = 453 (18.5%) HIV seropositive DBS specimens, with the following distribution: *n* = 67 (14.9%), *n* = 89 (19.8%), and *n* = 294 (65.3%) from GBMSM who used physical hotspots, virtual spaces, or a combination of both respectively to find male sex partners (*n* = 3 participants did not provide a response). Partial HIV *pol* sequences were obtained from *n* = 36 (10.6%) HIV seropositive DBS, of whom *n* = 6 (16.7%) primarily sought male sex partners online, *n* = 9 (25.0%) primarily sought male sex partners at physical hotspots, and *n* = 21 (58.3%) sought male sex partners online and at physical hotspots. Thus, only a small proportion of HIV seropositive DBS specimens were successfully sequenced, likely due to most study participants being virally suppressed (≤ 1,000 HIV RNA copies/mL; *n* = 298/341; 87.4%). Of these 36 sequences, the majority were classified as sub-subtype A1 (*n* = 21; 58.3%), followed by D (*n* = 4; 11.1%), A1D recombinants (*n* = 4; 11.1%), C (*n* = 3; 8.3%), A1C recombinants (*n* = 2; 5.6%), B (*n* = 1; 2.8%), and G (*n* = 1; 2.8%). An overview of the study workflow is depicted in Fig. 1.

**Fig. 2:**
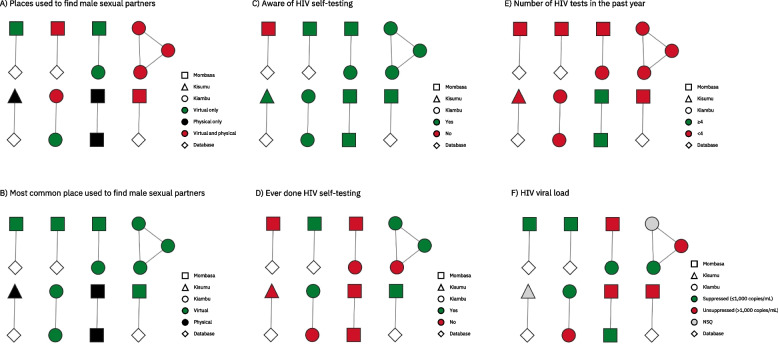
HIV phylogenetic clusters inferred using a maximum likelihood approach. HIV phylogenetic clusters were inferred by using a combination of node support and patristic distances. Clusters were defined as having a patristic distance ≤ 0.02 nucleotide substitutions per site. Each node represents an individual HIV *pol* sequence, while edges represent patristic distances ≤ 0.02 nucleotide substitutions per site. Maximum likelihood phylogenetic trees were reconstructed with IQ-TREE v1.6.12 using the GTR + F + I + G4 nucleotide substitution model. Clusters were visualized and annotated in Cytoscape v3.9.1. The abbreviation NSQ represents non-sufficient quantity for HIV viral load testing. HIV pol sequencing was prioritized over HIV RNA quantification. Consequently, for some of the 36 participants with available HIV pol sequences, viral load measurements were missing due to insufficient sample quantity remaining after sequencing. National guidelines in Kenya recommend quarterly HIV testing for key populations

Among the *n* = 36 HIV *pol* sequences from GBMSM included in our analysis, *n* = 13 (36.1%) were linked with one of eight distinct HIV phylogenetic clusters (Fig. 2, Supplementary File 3–9). All clusters were dyads, except for one cluster which contained three sequences. Overall, basic sociodemographic characteristics, sexual behaviors, and engagement with HIV prevention programs from GBMSM were similar among singleton and clustered HIV sequences (Table 1). However, a greater proportion of clustered sequences versus singleton sequences were from Kiambu county (*n* = 6/13 [46.2%] vs. *n* = 7/23 [30.4%]), reported using virtual hotspots exclusively to find male sex partners (*n* = 4/13 [30.8%] vs. *n* = 2/23 [8.7%]), and visited a government facility for their most recent HIV test (*n* = 8/13 [61.5%] vs. *n* = 6/22 [27.3%]). Furthermore, a smaller proportion of clustered sequences versus single sequences reported receiving money or gifts in exchange for sex with another man (*n* = 4/13 [30.8%] vs. *n* = 15/22 [68.2%]) and had a suppressed viral load (*n* = 5/9 [55.6%] vs. *n* = 13/17 [76.5%]). While a similar proportion of clustered (*n* = 3/22; 13.6%) and singleton (*n* = 2/13; 15.4%) HIV sequences were from participants who reported undergoing ≥ 4 HIV tests in the last year, all clustered sequences from participants reporting undergoing ≥ 4 HIV tests in the last year (*n* = 2/13; 15.4%) also reported using physical hotspots as the most common place to find male sex partners (Fig. 2B, 2E). In contrast, all clustered sequences from participants reporting undergoing < 4 HIV tests in the last year (n = 11/13; 84.6%) also reported virtual platforms as the most common place to find male sex partners (Fig. 2B, 2E).

**Table 1:** Sociodemographic characteristics of study participants

Variable	Singleton sequences (*n* = 23)		Clustered sequences (*n* = 13)		All PLWH (*n* = 453)	
	*n*	%	*n*	%	*n*	%
Age						
18–25 years	13	56.5	7	53.8	206	45.5
26–33 years	7	30.4	4	30.8	179	39.5
≥ 34 years	3	13.0	2	15.4	68	15.0
Marital status^a^						
Single	17	73.9	13	100.0	373	82.9
Married	2	8.7	0	-	51	11.3
Divorced/widowed/separated	4	17.4	0	-	26	5.8
County						
Kiambu	7	30.4	6	46.2	223	49.2
Kisumu	5	21.7	1	7.7	62	13.7
Mombasa	11	47.8	6	46.2	168	37.1
Self-identified sexual orientation^a^						
Gay	12	52.2	7	53.8	237	52.5
Bi-sexual	8	34.8	4	30.8	131	29.0
Heterosexual	3	13.0	2	15.4	77	17.1
Other	0	-	0	-	6	1.3
Self-identified sexual role/preference^a^						
Predominantly receptive (bottom)	8	34.8	5	38.5	130	29.0
Predominantly insertive (top)	4	17.4	2	15.4	108	24.1
Receptive and insertive (versatile)	11	47.8	6	46.2	211	47.0
Received money/gifts in exchange for sex with another man^a^	15	68.2	4	30.8	261	58.1
Places/locations used to find male partners						
Physical only	6	26.1	3	23.1	67	14.9
Virtual only	2	8.7	4	30.8	89	19.8
Physical and virtual	15	65.2	6	46.2	294	65.3
Most common place/location used to find male partners						
Physical	9	39.1	4	30.8	152	35.8
Virtual	14	60.9	9	69.2	272	64.2
Number of different male sexual partners in the past month^a^						
< 2	7	30.4	5	38.5	173	38.3
≥ 2	16	69.6	8	61.5	279	61.7
Condom used with last male sexual partner^a^	19	82.6	9	75.0	308	68.1
Ever tested for HIV^a^	22	95.7	13	100.0	439	97.3
Last place visited for most recent HIV test^a^						
Government facility	6	27.3	8	61.5	137	31.5
Private facility	2	9.1	2	15.4	45	10.3
MSM friendly clinic	10	45.5	2	15.4	202	46.4
Self-test	3	13.6	1	7.7	37	8.5
Other	1	4.5	0	-	14	3.2
Result of the most recent HIV test^a^						
Positive	10	45.5	5	38.5	181	42.0
Negative	12	54.5	8	61.5	247	57.3
Indeterminate	0	-	0	-	3	0.7
Number of HIV tests in the past year^a,b^						
< 4	19	86.4	11	84.6	269	77.7
≥ 4	3	13.6	2	15.4	77	22.3
Ever heard of HIV self-testing	20	87.0	11	84.6	395	87.2
Ever self-tested for HIV^c^	9	45.0	5	38.5	173	41.7
Ever been on ART^d^	8	100.0	5	100.0	154	93.3
Missed taking ARV drugs in the past month^d^	3	37.5	1	20.0	52	33.8
Suppressed viral load (≤ 1,000 copies/mL)^e^	13	76.5	5	55.6	298	87.4
Contacted by a peer educator/outreach worker in the past 3 months^a^	12	57.1	11	84.6	309	73.9
Visited a clinic or drop-in centre for MSM/MSW in the last three months	15	65.2	13	100.0	345	76.3
Ever registered with a program/NGO for MSM	17	73.9	8	61.5	353	79.0

Legend of the table: *n* = 453 *PLWH* (*n* = 23 singleton sequences, *n* = 13 clustered sequences, *n* = 417 without sequences). Overall, participants had a median age of 26 years (*IQR* = 23, 30), and the median participant age did not differ by sequence clustering condition.  *ART* anti-retroviral therapy, *ARV* anti-retroviral, *NGO* non-governmental organization, *MSM* men who have sex with men, *MSW* male sex worker

^a^Reflects the number and percentage of participants who answered this question

^b^National guidelines in Kenya recommend quarterly HIV testing for key populations

^c^Reflects the number and percentage of participants who are aware of HIV self-testing

^d^Reflects the number and percentage of participants who are aware of their HIV status and who answered this question

^e^Reflects the number and percentage of DBS specimens of sufficient quantity to perform HIV RNA quantification

Of the *n* = 36 participants for whom HIV *pol* sequences were available, *n* = 35 (97.2%) had previously tested for HIV. Among those who had ever tested and provided responses, *n* = 15 (42.9%) reported a positive result at their most recent HIV test, while *n* = 20 (57.1%) reported a negative result. This suggests that over half were unaware of their HIV-positive status at the time of the survey, even though they had reported testing previously. Notably, all participants (*n* = 13/13 [100.0%]; two participants did not respond) who were aware they were living with HIV at the time of the survey reported having ever been on antiretroviral therapy, with about one-third reporting having missed taking antiretroviral drugs in the past month (*n* = 4/13 [30.8%]). Among participants where enough DBS specimen was available for HIV RNA quantification, *n* = 8/26 (22.2%) were viremic (> 1,000 copies/mL) despite reporting ever being on antiretroviral therapy.

## Discussion

Our study aimed to describe HIV phylogenetic clusters among Kenyan GBMSM who use physical hotspots, online platforms, or a combination of both to find male sex partners. Despite the relatively small sample size, our analysis identified several HIV phylogenetic clusters representing approximately one-third of all specimens that were successfully sequenced. The majority of GBMSM within the HIV phylogenetic clusters mainly used virtual spaces to find male sex partners and did not receive money or gifts in exchange for sex with another man. Additionally, 84.6% of these individuals reported undergoing fewer than four HIV tests in the last year, despite most participants being aware of HIV self-testing, which may have contributed to overall low HIV viral load suppression (< 60%) among clustered GBMSM.

The rate of HIV phylogenetic clustering observed among GBMSM is consistent with other studies across countries in sub-Saharan Africa [30–33], Europe [34, 35], and Asia [36]. More specifically, between approximately 30% and 60% of HIV sequences from GBMSM formed HIV phylogenetic clusters depending on the study setting and patient recruitment strategy [32, 35]. These factors could influence the proportion of study participants who are virally suppressed, thereby potentially limiting the reconstruction of HIV transmission networks through phylogenetics. For example, studies like ours often oversample individuals actively engaged in HIV programmes for key populations [18], resulting in a sample with a high proportion of viral suppression. This may, in turn, lead to fewer available HIV sequences, as the sample underrepresents those not engaged in care and more likely to be viremic [37]. On the other hand, recruiting GBMSM who are not engaged in care and treatment may yield sample with a higher proportion of unsuppressed individuals, thereby producing a larger dataset of HIV sequences; yet, recruitment could be more challenging. While the clusters in our study consisted of two to three individuals, cluster size was likely influenced by the fact that only a small proportion of specimens were successfully sequenced. This presents a fragmented and biased snapshot of actual transmission networks. However, given the fragmentation, it is notable that there were any detectable phylogenetic linkages at all, which suggests high underlying connectivity and clustering within this population [38]. As well, it should be noted that small clusters have the potential to expand over time, especially if the prevention and treatment needs of those individuals are not met in a timely manner [39]. Other studies have highlighted the association between HIV phylogenetic clustering and HIV viremia [7, 40] as well as gaps in testing and linkage to care [7, 33, 40]. Despite the challenges in constructing more complete phylogenetic networks due to viral suppression, our findings do show connection within clusters by geography (counties) and methods for finding sexual partners. This suggests a not insubstantial degree of interaction between individuals in this population, by geography and partnering method; programmatically, this implies that prevention and intervention at the population-level cannot be siloed to specific groups. Thus, interventions should acknowledge this mixing, emphasizing the need for nimble HIV prevention and treatment interventions. Considering that GBMSM who use online platforms to find male sex partners are likelier not to be virally suppressed and test less frequently, the potential for expansion to similar members in mixing networks is concerning. Intervening and meeting the needs of individuals within these clusters, regardless of cluster size, is important to prevent further HIV transmission.

In our particular case, GBMSM in the HIV phylogenetic clusters mainly used virtual spaces (69.2%) to find male sex partners which has been associated with a higher risk of HIV acquisition due to several factors including HIV viremia [7, 40] and not being aware of their HIV status [13, 41]. Of particular concern, individuals within these clusters appeared to test for HIV less frequently (< 4 annual HIV tests) than recommended by national Kenyan guidelines for key populations [42]. The less-than-optimal testing frequency among GBMSM in clusters is a critical issue, as awareness and early detection of HIV infection are essential for initiating antiretroviral therapy, achieving viral suppression, and minimizing the risk of transmitting HIV [43]. Our study population is well connected to programs for GBMSM and male sex workers [18], yet the reported HIV testing frequency is unexpectedly low. This may indicate potential barriers to testing, such as service disruptions due to lockdowns during the COVID-19 pandemic [44]. Leveraging existing social networks and promoting HIV self-testing could enhance testing coverage and awareness of HIV status among GBMSM seeking male partners online [18], thereby preventing ongoing HIV transmission as suggested by our phylogenetic analysis. Previous work by our team suggests that once GBMSM living with HIV are diagnosed, initiation and adherence to antiretroviral therapy is generally good [45]. However, a non-negligible proportion of participants were viremic despite reporting ever being on antiretroviral therapy. COVID-19 restrictions led to interruptions in HIV treatment services, likely contributing to challenges in accessing antiretroviral drugs, maintaining adherence to therapy, and achieving viral suppression [46, 47].

Although understanding transmission at the population level is crucial, applying these insights to individual-level interventions raises significant ethical considerations (see for example: [48]). Directing interventions towards individuals identified within HIV phylogenetic clusters must be approached with caution to avoid exacerbating stigma, discrimination, and other potential harms [49]. Additionally, any use of HIV viral genomic data must be carefully balanced with privacy and human rights considerations to ensure the protection of those involved [50]. Indeed, our team is actively exploring both the possibilities and limitations of using HIV viral genomic data for key population programmes in Nairobi, Kenya, through a community-based participatory approach [44].

Despite the insights gained from our study, several limitations should be acknowledged. Given that only a small proportion of HIV seropositive DBS specimens could be sequenced, largely because most study participants were virally suppressed, our capacity to infer broader underlying transmission networks and investigate associations between behavioural data and HIV phylogenetic clustering was limited. The use of DBS specimens may have impacted our sequencing success rate, as recovery of HIV *pol* sequences from DBS specimens is generally less efficient than from serum or plasma, as reported by our team and by others, though still comparable overall [51–54]. Furthermore, nucleic acid degradation due to suboptimal storage conditions prior to analysis may have contributed to the low sequence recovery, as several viremic samples failed to generate HIV *pol* sequences. Nonetheless, we selected DBS for biological specimen collection in this study because they are generally well-received by research participants and offer several advantages over venipuncture, including ease of collection, storage, and transportation [55, 56]. The study focused solely on GBMSM data and samples from Kiambu, Kisumu, and Mombasa counties, thereby limiting the generalizability to other key populations and regions in Kenya. The use of face-to-face interviews may have introduced social desirability bias, potentially affecting the reporting of risk behaviours or HIV status. For instance, several participants who reported a negative result at their last HIV test were found to be virally suppressed, suggesting that many were likely on antiretroviral therapy but were uncomfortable disclosing their HIV status to interviewers due to fear of stigma or discrimination [45]. While we cannot completely rule out the possibility of data duplication, the participant selection process minimized this risk by ensuring that the same individuals were less likely to be included in both the baseline and endline surveys. Additionally, the distinct sociodemographic characteristics and varied survey responses among participants in HIV phylogenetic clusters further supported the likelihood that the sequences originated from different individuals. Nevertheless, our study provides important information on HIV transmission dynamics within GBMSM populations in Kenya and underscores the importance of targeted interventions to address specific risk profiles and enhance HIV prevention efforts.

## Conclusions

The findings of our study shed light on the dynamics of HIV transmission within GBMSM in Kenya. The observed HIV phylogenetic clustering, along with limited HIV testing rates among individuals in these clusters, necessitates interventions to prevent further transmission. HIV self-testing has shown promise in raising testing rates and awareness of HIV status among GBMSM, while network-based approaches could capitalize on existing social connections to reach individuals who may be missed by formal outreach efforts. By addressing these challenges and tailoring interventions to the specific needs of individuals, significant strides in controlling the HIV epidemic and improving overall public health outcomes in Kenya can be made.

## Supplementary Information

Supplementary Material 1.

Supplementary Material 2.

Supplementary Material 3.

Supplementary Material 4.

Supplementary Material 5.

Supplementary Material 6.

Supplementary Material 7.

Supplementary Material 8.

Supplementary Material 9.

## Data Availability

Sex work and same sex relationships are criminalized in Kenya. Therefore, we carefully considered the potential benefits and harms of using molecular data for GBMSM involved in our study. Based on discussions between members of community-based organisations in Kenya and the study team at the University of Manitoba, we have chosen not to publicly release our sequencing data to minimize any social harm resulting from our study. However, a request to access our data can be addressed to the corresponding author (FC).

## Abbreviations

COVID-19: Coronavirus disease 2019
DBS: Dried blood spot
GBMSM: gay, bisexual, and other men who have sex with men
HIV: Humane immunodeficiency virus
IQR: Interquartile range
NSQ: Non sufficient quantity
RT-PCR: Reverse transcription polymerase chain reaction
UFBoot: Ultra fast boostrap
