# Supplementary material for: HIV phylogenetic clusters point to unmet hiv prevention, testing and treatment needs among men who have sex with men in kenya
Source: BMC Infect Dis. 2024 Nov 20;24:1323. doi: 10.1186/s12879-024-10052-5 (PMC11580190; doi:10.1186/s12879-024-10052-5)
Supplement: Supplementary file 1 — Supplementary Material 1. [file 12879_2024_10052_MOESM1_ESM.docx]

**Annexure 2: Survey questionnaire for baseline and end line survey**

**Designing a community-based programmatic research to evaluate HIV Self-Testing among MSM in Kenya**

*FORM NO*

| SITE NAME AND CODE: ______________________________________________________________  DATE: ______________________________________________________  TYPE OF SITE: PHYSICAL VIRTUAL  TYPOLOGY: ___________________________  COUNTY: ______________________________________________SUB-COUNTY_______________________________________  INTERVIEWER NAME: __________________________________________________ SIGNATURE: _____________________________ _  SUPERVISOR NAME:___________________________________________________ SIGNATURE:__________________________________ |
| --- |

*1. Street; 2. Home; 3. Bus/taxi/truck stand; 4. Bar/restaurant; 5. Lodge/hotel; 6. Massage parlor; 7. Markets. 8. Through middleman; 9. Social gatherings; 10. Phone/mobile; 11. Internet; 12. Facebook; 13. Whatsapp; 14. Other (specify)­­­­…………………*

| **Sl** | **QUESTION** | **CATEOGRY** | **SKIP** |
| --- | --- | --- | --- |
| 1 | How old are you? | AGE IN COMPLETED YEARS |  |
| 2 | Are you currently studying? | YES 1  NO 0  NO ANSWER 99 |  |
| 3 | What is the highest level of education you attended/attending? | NO FORMAL EDUCATION 1  PRIMARY 2  SECONDARY 3  TERTIARY/COLLEGE/UNIVERSITY 4  OTHERS (SPECIFY) 97  NO ANSWER 99 |  |
| 4 | What is your marital status? | SINGLE 1  MARRIED 2  DIVORCED/WIDOWED/SEPARATED 3  NO ANSWER 99 |  |
| 5 | Who are you currently living with?  MORE THAN ONE OPTION POSSIBLE | LIVING ALONE………………………………. A  PARENTS………………..…………………… B  BROTHER(S) AND/OR SISTER….……………. C  OTHER FAMILY……………………………. D  MALE SEXUAL PARTNER……………………………. E  FEMALE SEXUAL PARTNER……………………... F  CHILDREN…………………… ……………………. G  OTHERS (SPECIFY) X  NO ANSWER ……………………………… Z |  |
| 6 | What is your main occupation/employment? | BUSINESS 1  CONSTRUCTION WORKER 2  FACTORY WORKER 3  FARMING/AGRICULTURAL WORKER 4  FISHERMAN/SEAFARER 5  GOVERNMENT WORKER 6  HAIRDRESSER/BEAUTICIAN/MASSEUSE 7  SHOP WORKER 8  STREET VENDOR/CASUAL LABOURER 9  TOURISM/TRAVEL AGENT/TOUR GUIDE 10  WAITER-WAITRESS/BARTENDER/  HOTEL EMPLOYEE 11  SEX WORK 12  NO INCOME 13  OTHER (SPECIFY): 97  DON’T KNOW 98  NO ANSWER 99 | 8 |
| 7 | What is your average monthly income? | SHILLING |  |
| 8 | In which County / Sub county do you live? | COUNTY: _______________________________________________________  SUBCOUNTY:__________________________________________________________ |  |
| 9 | How do you predominantly describe your sexual orientation/identity? | GAY 1  BI SEXUAL 2  MSM 3  MSW 4  OTHER (SPECIFY): 97  NO ANSWER 99 |  |
| 10 | How do you describe your sexual preference/role? | PREDOMINANTLY RECEPTIVE (BOTTOM/HANITHI) 1  PREDOMINANTLY INSERTIVE (TOP/BASHA) 2  BOTH RECEPTIVE AND INSERTIVE (VERSATILE) 3  OTHERS (SPECIFY) 97  NO ANSWER 99 |  |
| 11 | How old were you when you had your first sex? | AGE  NEVER HAD SEX ………….95  DON’T KNOW………………………… 98  NO ANSWER………………………… 99 | END |
| 12 | How old were you when you had you had first anal or oral sex with a man? | AGE  NEVER HAD ANAL SEX ………….95  DON’T KNOW………………………… 98  NO ANSWER………………………… 99 | END |
| 13 | In the last 3 months, how many different male sexual partners have you had? | NUMBER OF PARTNERS  NONE 00  DON’T KNOW…………. 98  NO ANSWER………………………… 99 | END |
| 14 | In the last 1 month, how many different male sexual partners have you had? | NUMBER OF PARTNERS  NONE 00  DON’T KNOW…………. 98  NO ANSWER………………………… 99 | END |
| 15 | How many different male sexual partners have you had in the last one week? | NUMBER OF PARTNERS  NONE 00  DON’T KNOW…………. 98  NO ANSWER………………………… 99 |  |
| 16 | How many sex acts have you had in the last one week? | NUMBER OF SEX ACTS  NONE 00  DON’T KNOW…………. 98  NO ANSWER………………………… 99 |  |
| 17 | During the period of COVID-19 (specify months), was there a change in the number of male sexual partners you had per week? | INCREASED 1  DECREASED 2  REMAINED MORE OR LESS THE SAME 3  DON’T KNOW…………. 98  NO ANSWER………………………… 99 |  |
| 18 | During the period of COVID-19 (specify months), on an average, how many different male sexual partners you had in a week? | NUMBER OF PARTNERS  NONE 00  DON’T KNOW…………. 98  NO ANSWER………………………… 99 |  |
| 19 | The last time when you had sex with a man, were you both aware of each other’s HIV status? | NO 0  YES 1  I WAS AWARE BUT PARTNER WAS NOT 2  PARTNER WAS AWARE AND I WAS NOT 3  NO ANSWER 99 |  |
| 20 | Do you receive money/gifts in exchange of sex with another man? | YES 1  NO 0  NO ANSWER 99 |  |
| 21 | Do you charge for sex? | YES 1  NO 0  NO ANSWER 99 |  |
| 22 | Which are the different places/locations through which you have met other male sexual partners?  MORE THAN ONE OPTION POSSIBLE | STREET…………………………. A  HOME…………………………. B  BUS/TAXI/TRUCK STAND…………. C  BAR/RESTAURANT....................... E  LODGE/HOTEL…………………… F  MASSAGE PARLOR………………. G  MARKETS………………………… H  THROUGH MIDDLEMAN…………. I  SOCIAL GATHERINGS……………. J  PHONE/MOBILE………………… K  INTERNET/WEBAPP………………………… L  FACEBOOK M  WHATSAPP N  OTHER (SPECIFY)­­­­………………… X  NO ANSWER…………………………… Z |  |
| 23 | In the last one month, which are the different places/locations through which you have met other male sexual partners? | STREET…………………………. A  HOME…………………………. B  BUS/TAXI/TRUCK STAND…………. C  BAR/RESTAURANT....................... E  LODGE/HOTEL…………………… F  MASSAGE PARLOR………………. G  MARKETS………………………… H  THROUGH MIDDLEMAN…………. I  SOCIAL GATHERINGS……………. J  PHONE/MOBILE………………… K  INTERNET………………………… L  FACEBOOK M  WHATSAPP N  OTHER (SPECIFY)­­­­………………… X  NO ANSWER…………………………… Z |  |
| 24 | Among the above places, which is the most common place through which you meet most male sexual partners? | CODE THE CORRESPONDING FROM ABOVE |  |
| 25 | During the period of COVID-19 period (specify months), what were the different places/locations through which you met other male sexual partners? | STREET…………………………. A  HOME…………………………. B  BUS/TAXI/TRUCK STAND…………. C  BAR/RESTAURANT....................... E  LODGE/HOTEL…………………… F  MASSAGE PARLOR………………. G  MARKETS………………………… H  THROUGH MIDDLEMAN…………. I  SOCIAL GATHERINGS……………. J  PHONE/MOBILE………………… K  INTERNET………………………… L  FACEBOOK M  WHATSAPP N  OTHER (SPECIFY)­­­­………………… X  NO ANSWER…………………………… Z |  |

| 26 | Now I would like to ask you few questions about the 2 most recent male sexual partners you had; | | | |
| --- | --- | --- | --- | --- |
| S.N |  | MOST RECENT PARTNER | SECOND MOST RECENT PARTNER |  |
| A | Where did you meet him for the first time?  **USE CODES FROM QUESTION 21** |  |  |  |
| B | When you first had sex with this partner, how old were you? | Age | Age |  |
| C | For how long have you had/ having sexual relations with this partner? | MONTHS 1  YEARS 2  DON’T KNOW 98 | MONTHS 1  YEARS 2  DON’T KNOW 98 |  |
| D | In the past 30 days, how many times have you had sex with this partner? | NO. OF TIMES | NO. OF TIMES |  |
| E | The last time when you had sex with this partner, was a condom used? | YES 1  NO 0  DON’T KNOW 98  NO ANSWER 99 | YES 1  NO 0  DON’T KNOW 98  NO ANSWER 99 |  |
| F | What motivated you to have sex with this person? | MONEY 1  PLEASURE 2  OTHERS (SPECIFY) 97  NO ANSWER 99 | MONEY 1  PLEASURE 2  OTHERS (SPECIFY) 97  NO ANSWER 99 |  |
| G | Did you pay money/gifts/favors when you had sex last time with this person? | YES, MONEY 1  YES, GIFT 2  NO 3  NO ANSWER 99 | YES, MONEY 1  YES, GIFT 2  NO 3  NO ANSWER 99 |  |
| H | Did you receive money/gifts/favors when you had sex last time with this person? | YES, MONEY 1  YES, GIFT 2  NO 3  NO ANSWER 99 | YES, MONEY 1  YES, GIFT 2  NO 3  NO ANSWER 99 |  |

| **Sl** | **QUESTION** | **CATEGORY** | **SKIP** |
| --- | --- | --- | --- |
| 27 | The last time when you had sex with any of your male partner, was a condom used? | YES 1  NO 0  NO ANSWER…………………………… 99 | 29 |
| 28 | Why didn’t you use a condom with that partner? | MORE PLEASURE/ I PREFER BARE BACK A  IN LOVE WITH THE PARTNER B  LOSS OF ERECTION C  AWARE OF PARTNER’S HIV STATUS D  CONDOM WAS NOT AVAILABLE E  CONDOM BROKE OR SLIPPED OFF F  ALCOHOL AND/ OR DRUG USE G  RESISTANCE FROM PARTNER H  MUTUAL AGREEMENT I  FAMILIARITY, TRUST AND RECIPROCITY J  HEAT OF THE MOMENT K  DIDNT WANT TO USE A CONDOM L  CONDUCTED HIV SELT TESTING TOGETHER M  OTHERS (SPECIFY) X  NO ANSWER…………………………… Z |  |

**HIV TESTING**

| **Sl** | **QUESTION** | **CATEOGRY** | **SKIP** |
| --- | --- | --- | --- |
| 29 | Do you know a place where HIV testing can be done? | YES 1  NO 0  DON’T KNOW 98  NO ANSWER 99 | 31 |
| 30 | Which is/are the place/s where you can visit for HIV testing?  **MORE THAN ONE OPTION POSSIBLE** | GOVERNMENT FACILITY A  PRIVATE FACILITY B  MSM FRIENDLY CLINICS C  HIV SLEFTESTING AT HOME……………………………………… D  OTHERS(SPECIFY) X  NO ANSWER Z |  |
| 31 | Have you ever been tested for HIV? | YES 1  NO 0  DON’T KNOW 98  NO ANSWER 99 | 45 |
| 32 | How many years/months back did you take your first HIV test? | YEARS AGO  MONTHS AGO  DON’T KNOW 98  NO ANSWER 99 |  |
| 33 | How many months after your first sex, did you take your first or repeat (if taken HIV test before your first sex) HIV test? | NUMBER OF MONTHS AFTER FIRST SEX    NEVER TAKEN AN HIV TEST AFTER FIRST SEX 97  DON’T KNOW………………………… 98  NO ANSWER………………………… 99 |  |
| 34 | How many times have you tested for HIV in the last 12 months? | NUMBER OF TIMES  NONE 00  DON’T KNOW 98  NO ANSWER 99 |  |
| 35 | When did you have your MOST recent HIV test? | NUMBER OF MONTHS AGO  DON’T KNOW 98  NO ANSWER 99 |  |
| 36 | Which was the place where you took your most recent HIV test? | GOVERNMENT FACILITY 1  PRIVATE FACILITY 2  MSM FRIENDLY CLINICS 3  SELF TEST 4  OTHERS (SPECIFY): 97  NO ANSWER 99 |  |
| 37 | What was the test result of your most recent HIV test? | HIV-POSITIVE 1  HIV-NEGATIVE 2  INDETERMINATE 3  DID NOT RECEIVE RESULT 4  DON’T KNOW 98  NO ANSWER 99 |  |
| 38 | During the past 12 months, did you have an occasion where you wanted to test for HIV, but did not? | YES 1  NO 0  NO ANSWER 99 | 40 |
| 39 | What was the reason for not testing? | COULD NOT GO FOR TESTING  DUE TO COVID-19 A  HIV TESTING FACILITY WAS NOT AVAILABLE B  HIV SELF TESTING KIT WAS NOT AVAILABLE C  I DON’T THINK I HAVE HIV/NO REASON TO TEST D  AFRAID OF LEARNING HIV STATUS E  LACK OF CONFIDENTIALITY F  TAKES TOO MUCH TIME G  NOT AWARE OF A PLACE H  OTHERS (SPECIFY): X  NO ANSWER Z |  |
| 40 | During COVID-19 period (specify months) did you take an HIV test? | DUE FOR HIV TEST AND TESTED 1  DUE FOR HIV TEST AND NOT TESTED 2  NOT DUE FOR HIV TEST 3  WAS ALREADY LIVING POSITIVELY 4  NO ANSWER 99 | 42 |
| 41 | During COVID-19 period (specify months), what type of HIV test did you undertake? | HIV SELF-TESTING 1  UNIVERSAL HIV TESTING 2  BOTH (SELF-TESTING &UNIVERSAL) 3  DID NOT DO HIV TESTING 4  WAS ALREADY LIVING POSITIVELY 5  NO ANSWER 99 |  |
| 42 | Have you ever been initiated on PrEP? | YES 1  NO 0  NO ANSWER 99 | 45 |
| 43 | Are you currently taking PrEP? | NO 0  YES 1  LIVING POSITIVELY 2  NO ANSWER 99 |  |
| 44 | During COVID-19 period (specify months), did you get your PrEP medication? | YES, IT DID’NT AFFECT MY ABILITY TO GET PREP……….1  YES, I STILL GOT IT BUT IT WAS MORE DIFFICULT TO GET  …... 2  NO, I STOPPED PREP AS I COULD NO LONGER  GET IT ………3  NO, THERE WAS NO RISK BEHAVIOUR SO I DID NOT  TAKE IT ……………………………………………………………………..4  LIVING POSITIVELY……. .. 5  DON’T KNOW 98  NO ANSWER 99 |  |
| 45 | Are you enrolled in any care and treatment center (CCC)? | NO 0  YES 1  NEVER TESTED POSITIVE 2  NO ANSWER 99 | 51 |
| 46 | How long have been enrolled in any care and treatment center (CCC)? | NUMBER OF MONTHS AGO |  |
| 47 | Have you ever been on ART? | YES 1  NO 0  NO ANSWER 99 | 49 |
| 48 | How long have you been on ART? | NUMBER OF MONTHS |  |
| 49 | Have you ever missed taking ARV in the past one month? | YES 1  NO 0  NO ANSWER 99 |  |
| 50 | During COVID-19 period (specify months), did you get your ARV? | YES, I COULD GET ARV THE SAME AS BEFORE …………..1  YES, I COULD GET ARV BUT IT WAS MORE DIFFICULT  TO FIND 2  I COULD NOT GET ARV 3  DON’T KNOW 98  NO ANSWER 99 |  |

|  | **BEFORE ASKING QUESTION 51, THE INTERVIEWER TO EXPLAIN WHAT HIV-SELF TESTING IS IN DETAILS** | |  |
| --- | --- | --- | --- |
| Sl | QUESTION | CATEGORY | SKIP |
| 51 | Have you heard of HIV self-testing? | YES 1  NO 0  DON’T KNOW 98  NO ANSWER 99 | 64 |
| 52 | How did you get to hear about HIV Self-testing? | PEER EDUCATOR/OUTREACH WORKER A  SOCIAL MEDIA B  HEALTH CARE PROVIDER C  RADIO/TV D  CLUB E  MSM PARTY F  ROAD SHOWS G  OTHERS (SPECIFY): X  NO ANSWER Z |  |
| 53 | How many month ago was the first time when you ever heard of HIV self-testing? | NUMBER OF MONTHS AGO  DON’T KNOW 98  NO ANSWER 99 |  |
| 54 | Have you ever used HIV Self-Testing ? | YES 1  NO 0  DON’T KNOW 8  NO ANSWER 9 | 64 |
| 55 | How many months ago was the first time when used HIV self-testing? | NUMBER OF MONTHS AGO  DON’T KNOW 98  NO ANSWER 99 |  |
| 56 | The last time when you used HIV self-testing, how many months ago was it? | MONTHS AGO |  |
| 57 | In the past 12 months , how many times did you test for HIV using HIV self-testing ? | ONCE IN 3 MONTHS 1  ONCE IN 6 MONTHS 2  ONCE IN A YEAR 3  DON’T KNOW 98  NO ANSWER 99 |  |
| 58 | This last time when you used HIV self-testing, was it repeat or first time? | FIRST TIME 1  REPEAT………………………………………………………………………2  NO ANSWER 99 |  |
| 59 | The last time when you used HIV self-testing, where did you test? | HOME 1  FRIENDS PLACE 2  MSM FRIENDLY CLINIC 3  GOVERNMENT FACILITY 4  PRIVATE FACILITY 5  HOTSPOT ………………………………….………………………………6  OTHERS (SPECIFY): 97  NO ANSWER 99 |  |
| 60 | The last time when you used HIV self- testing, where did you receive the test kit? | PHARMACY 1  FRIENDS 2  MSM FRIENDLY CLINIC 3  GOVERNMENT FACILITY 4  PRIVATE FACILITY 5  PEER EDUCATOR 6  OUTREACH WORKER 7  OTHERS (SPECIFY) 97  NO ANSWER 99 |  |
| 61 | The last time when you used HIV self-testing, who assisted you for testing? | PARTNER 1  PEER EDUCATOR/ OUTREACH WORKER 2  COUNSELOR 3  HEALTH CARE PROVIDER 4  SELF/NO ASSISTANCE 5  OTHERS (SPECIFY) 97  NO ANSWER 99 |  |
| 62 | During the COVID-19 period (specify months), did you get HIV self-test kits? | YES, I COULD GET HIV SELF-TEST SAME AS BEFORE….1  YES, I COULD GET HIVST BUT IT WAS MORE  DIFFICULT TO FIND………………………………………………….. 2  NO, I COULD NO LONGER GET AN HIV SELF-TEST 3  DON’T KNOW 98  NO ANSWER 99 |  |
| 63 | In the last 3 months, was there an occasion when you wanted to use HIVST but could not find? | NO 1  YES, BECAUSE OF COVID-19 2  YES, DIDNT KNOW WHERE TO FIND ONE 3  YES, DIDNT HAVE THE MONEY TO BUY ONE 4  OTHERS (SPECIFY): 97  NO ANSWER 99 |  |
| 64 | The next time when you go for HIV testing, which place would you go? | GOVERNMENT FACILITY 1  PRIVATE FACILITY 2  MSM FRIENDLY CLINICS 3  HIV SELF-TEST 4  LIVING POSITIVELY 5  OTHERS (SPECIFY): 97  NO ANSWER 99 |  |
| 65 | The next time when you go for HIV testing, which method would you go? | HIV SELF-TESTING 1  UNIVERSAL HIV TESTING 2  LIVING POSITIVELY 3  NO ANSWER 99 |  |
| 66 | According to you what are the advantages of using HIV self-testing?  **MORE THAN ONE RESPONSE** | PRIVACY A  CONVENIENCE B  NOONE KNOWS MY STATUS C  NO BLOOD SAMPLE/PRICK REQUIRED D  NO NEED TO VISIT HEALTH FACILITY E  CAN DO AT HOME/AT CONVENIENCE F  NO ADVANTAGE G  OTHERS (SPECIFY): X  DO NOT KNOW…………………………………………………………Y  NO ANSWER Z |  |
| 67 | What do you think are the main disadvantages of HIV self-testing?  **MORE THAN ONE OPTION POSSIBLE** | NO DISADVANTAGE A  LACK OF POST TEST CONSELING INCREASE  DISTRESS B  POSSIBILITY OF SELF-HARM C  POSSIBILITY OF HARMING OTHERS D  REDUCED CHANCE OF DISCLOSURE/  ENROLLMENT IN CARE E  OTHERS (SPECIFY) X  DO NOT KNOW………………………………………………………… Y  NO ANSWER Z |  |
| 68 | How would you like to receive HIV self-test kits?  **MORE THAN ONE OPTION POSSIBLE** | PEER EDUCTOR/ORW A  HTS B  NGO/PROGRAMS C  PHARMACY D  FRIENDS E  OTHERS (SPECIFY) X  NO ANSWER Z |  |

**PROGRAM EXPOSURE**

| 69 | Have you ever heard of an organization/NGO in this County offering health services for men who have sex with men? | YES 1  NO 0  DON’T KNOW 98  NO ANSWER 99 | **71** |
| --- | --- | --- | --- |
| 70 | How many months ago was the first time you heard of an organization/NGO in this County offering health services for men who have sex with men? | NUMBER OF MONTHS AGO  DON’T KNOW 98  NO ANSWER 99 |  |
| 71 | Have you ever been contacted by a peer educator/outreach worker? | YES 1  NO 0  DON’T KNOW 98  NO ANSWER 99 | **77** |
| 72 | The first time when you were contacted by a peer educator/outreach worker, how many months ago was it? | NUMBER OF MONTHS AGO  DON’T KNOW 98  NO ANSWER 99 |  |
| 73 | In the last 3 months, were you contacted by a peer educator/outreach worker? | YES 1  NO 0  DON’T KNOW 98  NO ANSWER 99 |  |
| 74 | The last time when you were contacted by a peer educator/outreach worker, how/where did he contact you? | AT HOME 1  AT PHYSICAL LOCATION OF MSM 2  THROUGH PHONE 3  VIRTUAL SITE 4  OTHER (SPECIFY): 97  NO ANSWER 99 |  |
| 75 | The last time when you were contacted by a peer educator/outreach worker, what services did you receive? | CONDOM A  LUBRICANTS B  HIV/STI EDUCATION C  HIV SELF TESTING D  VIOLENCE SUPPORT………………………………………………….E  COVID 19 RELATED EDUCATION………………………………..F  PPE (MASK, SANITIZERS)…………………………………………..G  REFERRAL TO HEALTH FACILITY………………………………..H  OTHERS (SPECIFY): X  NO ANSWER Z |  |
| 76 | During the period of COVID-19 (specify months), did you meet a peer educator? | YES, I MET LIKE OTHER MONTHS 1  YES, BUT VERY INFREQUENCTLY 2  NO, I COULD NOT MEET……………………………………………3    DON’T KNOW 98  NO ANSWER 99 |  |
| 77 | Which source/s would you like to receive HIV related information/services? | PEER EDUCATOR/ORW A  INTERNET/WEBSITE B  PHONE C  ELECTRONIC/PRINT MEDIA D  OTHERS (SPECIFY): X  NO ANSWER Z |  |
| 78 | Have you ever visited a clinic or drop-in-center in or around your town that provides health information or services to MSM/MSW? | YES 1  NO 0  DON’T KNOW 98  NO ANSWER 99 | 85 |
| 79 | The first time when you visited a clinic or drop-in-center in or around your town that provides health information or services to MSM/MSW, how many months ago was it? | NUMBER OF MONTHS AGO  DON’T KNOW 98  NO ANSWER 99 |  |
| 80 | Have you ever visited a clinic or drop-in Centre in or around your town that provides health information or services to MSM/MSW in the past 3 months? | YES 1  NO 0  DON’T KNOW 98  NO ANSWER 99 |  |
| 81 | In the past 6 months, was there an occasion when you wanted to visit a drop-in-center, but did not visit? | YES 1  NO 0  DON’T KNOW 98  NO ANSWER 99 | 83 |
| 82 | What was the reason for not visiting the DIC in the past 6 months? | NOT ABLE TO TRAVEL DUE TO COVID-19 1  DIC WAS NOT OPEN 2  DID NOT GET AN APPOINTMENT 3  DID NOT HAVE MONEY 4  OTHERS (SPECIFY) 97  NO ANSWER 99 |  |
| 83 | During the period of COVID-19 (specify months), did you visit the clinic or drop-in centre? | YES, IT DID NOT AFFECTED MY ABILITY TO VISIT A CLINIC/DROP-IN CENTRE 1  YES, BUT IT WAS MORE DIFFICULT TO VISIT A CLINIC/  DROP-IN CENTRE 2  NO, I COULD NO LONGER VISIT A CLINIC/DROP-IN CENTRE…... 1  DON’T KNOW 98  NO ANSWER 99 |  |
| 84 | The last time when you visited a clinic/drop-in-center, what service/s did you receive? | CONDOM A  LUBRICANTS B  STI SCREENING C  STI TREATMENT D  HIV TESTING E  HIV SELF-TESTING KIT F  COUNSELING G  PrEP………………………………………………………………………….H  ART……………………………………………………………………………I  OTHERS (SPECIFY) X  NO ANSWER Z |  |
| 85 | Have you ever registered with a program/NGO that provides exclusive services to MSM community? | YES 1  NO 0  DON’T KNOW 98  NO ANSWER 99 |  |

**ALCOHOL, DRUG AND VIOLENCE AND STIGMA**

| 86 | During the past month, how often have you consumed drinks containing alcohol? | NEVER…………………………………………… 1  EVERY DAY…………………………… 2  AT LEAST ONCE A WEEK………………… 3  LESS THAN ONCE A WEEK………………… 4  NOT IN THE PAST MONTH………………… 5  DON’T KNOW………………………… 98  NO ANSWER……………………………………… 99 |  |
| --- | --- | --- | --- |
| 87 | In the last 3 months, how often were you or your clients/partner under the influence of alcohol while having sex? | EVERYTIME………………………………… 1  OFTEN…………………………………………… 2  SOMETIME……………………………………… 3  NEVER…………………………… 97  NO ANSWER……………………………………… 99 |  |
| 88 | During the period of COVID-19 (specify months), was there a change in your alcohol drinking behavior in comparison to other months? | INCREASED 1  DECREASED 2  REMAINED MORE OR LESS THE SAME 3  DON’T KNOW…………. 98  NO ANSWER………………………… 99 |  |
| 89 | Some people consume drugs for no-medical reasons, like marijuana, heroin, amphetamine, etc to feel good, get high, fly, trip or fantasies. Have you ever consumed drugs like these? | YES………………………… 1  NO……………………………………… 0  DON’T KNOW…………………………………… 98  NO ANSWER…………………………………… 99 |  |
| 90 | Have you ever injected drugs for non-medical reasons? | YES………………………… 1  NO……………………………………… 0  DON’T KNOW…………………………………… 98  NO ANSWER…………………………………… 99 | 93 |
| 91 | In the past 12 months, have you ever injected drugs for non-medical reasons? | YES………………………… 1  NO……………………………………… 0  DON’T KNOW…………………………………… 98  NO ANSWER…………………………………… 99 |  |
| 92 | When you injected drugs in the past 12 months, did you share needle with any one? | YES 1  NO 0  DON’T KNOW 98  NO ANSWER 99 |  |
| 93 | During the period of COVID-19 (specify months), did your drug use behavior change compared to other months? | INCREASED 1  DECREASED 2  REMAINED MORE OR LESS THE SAME 3  I DON’T DO DRUGS 4  DON’T KNOW…………. 98  NO ANSWER………………………… 99 |  |
| 94 | In the past 12 months, have you been verbally or physically assaulted/abused by family/ community? | YES 1  NO 0  DON’T KNOW 98  NO ANSWER 99 | 96 |
| 95 | The last time you experienced a verbal or physical assault/abuse, who perpetrated it? | PARTNER 1  FAMILY MEMBERS 2  FRIENDS 3 COLLEAGUES 4  NEIGHBOURS 5  POLICE/LAW ENFORCEMENT 6  OTHERS (SPECIFY) 97  NO ANSWER 99 |  |
| 96 | In the past 12 months, have you been sexually assaulted/abused by someone? | YES 1  NO 0  DON’T KNOW 98  NO ANSWER 99 | 98 |
| 97 | The last time you experienced a sexual assault/abuse, who perpetrated it? | PARTNER 1  FAMILY MEMBERS 2  FRIENDS 3 COLLEAGUES 4  NEIGHBOURS 5  POLICE/LAW ENFORCEMENT 6  OTHERS (SPECIFY) 97  NO ANSWER 99 |  |
| 98 | In the past 12 months, have you ever been excluded from social gatherings/family/ friends/community? | YES 1  NO 0  DON’T KNOW 98  NO ANSWER 99 | 100 |
| 99 | The last time you experienced an exclusion from social gatherings/family/friends/ community, who perpetrated it? | FAMILY MEMBERS 1  FRIENDS 2 COLLEAGUES 3  NEIGHBOURS 4  POLICE/LAW ENFORCEMENT 5  OTHERS (SPECIFY) 97  NO ANSWER 99 |  |
| 100 | In the past 12 months, have you ever been rejected by health care provider because of HIV status? | YES 1  NO 0  DON’T KNOW 98  NO ANSWER 99 | 102 |
| 101 | The last time when you were rejected by a health care provider, what type of facility was it? | GOVERNMENT FACILITY 1  PRIVATE FACILITY 2  NGO RUN FACILITY 3  OTHERS (SPECIFY) 97  NO ANSWER 99 |  |
| 102 | In the past 12 months, have you ever harassed/arrested by a law enforcement agencies? | YES 1  NO 0  DON’T KNOW 98  NO ANSWER 99 |  |
| 103 | During the period of COVID-19 (specify months), did you experience violence from the police/Law enforcers/Authorities? | YES, SAME AS BEFORE………………………………………………1  YES, VIOLENCE INCREASED MORE THAN BEFORE 2  NO, I DID NOT EXPERIENCE VIOLENCE 3  NO ANSWER 99 |  |
| 104 | During the period of COVID-19 (specify months), did you experience violence from intimate partner? | YES, SAME AS BEFORE………………………………………………1  YES, VIOLENCE INCREASED MORE THAN BEFORE 2  NO, I DID NOT EXPERIENCE VIOLENCE 3  NO ANSWER 99 |  |

THANKS
