# Supplementary material for: HIV phylogenetic clusters point to unmet hiv prevention, testing and treatment needs among men who have sex with men in kenya
Source: BMC Infect Dis. 2024 Nov 20;24:1323. doi: 10.1186/s12879-024-10052-5 (PMC11580190; doi:10.1186/s12879-024-10052-5)
Supplement: Supplementary file 2 — Supplementary Material 2. [file 12879_2024_10052_MOESM2_ESM.docx]

**HIV subtype A1 background sequences (*n* = 1,934)**

MN791352, MN791337, MN791341, MN791339, MN791342, MN791336, MN791338, MN791358, MN791399, MN791364, MN791398, MN791397, MN791430, MN791401, MN791395, MN791393, MN791434, MN791371, MN791360, ON959680, KC517057, HQ993815, MH366886, KM016179, HQ993945, KF716475, MK192596, KC018726, KC018845, MK192609, MK192568, KC018640, KC018951, GQ400175, EF186206, MN791747, MN791736, MN791711, MN791717, MN791743, MN791728, MN791730, MN791741, MN791724, ON959701, JQ616940, KF716474, KY579585, KC568526, HQ993870, HQ993752, HQ993872, AF004885, OM110106, KX302419, KX302452, KX302453, KT022370, KC018969, KC018962, KC018963, KJ907060, KC517054, DQ136708, DQ136695, DQ136691, DQ136698, DQ136697, DQ136696, DQ136699, DQ136692, DQ136690, DQ136709, DQ136693, DQ136707, DQ136704, DQ136705, DQ136694, AY322185, AY322184, AF457070, HQ993790, KY579566, HQ993771, HQ993668, AF361872, EF368970, KY579445, KY579438, KX692205, GQ303792, MG435479, KJ907056, KJ907030, HQ993828, KC516998, KC516877, HQ993650, KX692215, KX692201, KX302495, KX302496, KX692024, MN791791, MN791796, MN791785, MN791780, MN791781, MN791786, MN791798, MN791792, JN132304, AJ286980, KC517016, HQ993770, HQ993798, HM164114, KX306434, KX306433, AY492761, EF612181, AY435385, KX306387, AY253314, KT022368, JX460024, KX306406, KX791048, JX460300, HQ993652, HQ993882, HQ993638, AF457066, JQ698384, KC568516, DQ079827, MN816768, KC568488, HM119717, EU110094, JQ616920, HQ993820, GQ400857, KU498356, HQ993932, KX306402, JQ480170, HQ993862, AY492780, KX306435, KX306436, KC516927, JQ616938, HQ993774, HQ993671, KM057341, MH354016, HQ993726, HQ993613, HQ993878, JQ616939, HQ993693, HQ993817, OM110082, MK513217, ON501445, HQ993628, KX302594, KX302582, KX302583, HQ993864, KX791013, HQ993910, HQ993695, HQ993804, HQ993793, HQ993959, AF457053, HQ993572, ON501501, JX123580, JX123655, KT152841, KT152840, KT318921, EU447782, EU158893, KT152842, HM002536, KJ185340, KJ185233, KJ185199, MH366880, KC516886, HQ993935, KU498330, HQ993575, AY492765, HQ441646, KM057329, HQ441742, AF457069, KC516932, ON501504, JN628473, JQ071458, MG435678, HQ993791, HQ993814, KJ906738, KC516884, AF457067, JN628526, EU251773, HQ993679, JQ698414, KX790996, KM016214, HQ993712, HQ993735, KX306381, HQ993850, JQ480190, HQ993923, KC516923, JQ625653, JN628488, JN628487, KX306457, KX302562, KX302561, KX302560, KX302559, HQ993742, FJ623486, KC019097, HQ993894, KC516990, AF457075, KC517031, KP877891, KX306378, KT370899, HQ993875, HQ441710, JQ698419, JQ698361, HQ441597, KC018770, HQ993608, HQ993647, ON501548, AF457077, OM109853, MH367001, OM110027, FJ623482, OM110073, MH366894, EU110087, EU251822, KJ907076, AF457086, AF410245, EF612176, EF612094, MG435855, JQ698403, KC841661, KC841670, HQ993700, JN628524, AY492753, KC516976, KJ907003, KC516964, OM110113, KR003390, JQ480179, KX306382, HQ993658, HQ993844, HQ993640, HQ993794, HQ993936, JX123626, EF186204, HQ993934, ON501448, ON501463, ON501508, MH317457, KC516908, HQ441595, FJ623478, HM164125, JN630901, KY579571, KX790986, JN628494, JQ625659, EU110092, EU110097, KX306424, JQ625616, JQ625605, KY579544, KC018519, ON501597, JX123622, JQ625649, KC516944, HQ993911, MT185163, KC517015, HQ993687, ON501453, HQ993957, MH509760, HQ993691, HQ993777, KX306397, KC516997, HQ993639, HQ993846, ON501556, JN630892, ON501575, JX460545, KC018918, KJ907165, KX692206, JQ616933, KC841777, KC516937, MG435458, OK624492, AF484512, JQ616942, HQ993835, KC517011, KC516965, OM109843, OM109840, KC517023, JN630909, MN240784, KP877914, OM109885, KC516909, JN630910, KP877930, JX460032, JX460267, KY579426, KX306379, ON501564, HQ993954, HQ441707, MH575299, JQ698375, HQ441663, JN628511, HQ993746, HQ993649, HQ993627, ON501606, HQ993919, HQ993686, KT022383, MH317488, KC018723, OM109822, OM109824, OM109823, MT185162, MT185271, KC018658, KC018904, KC018699, KC018587, OM109883, HM164122, KR138543, HM164130, ON501547, MG435463, KT833393, KT833478, KX306439, KX306391, KP877901, KT370919, HQ441636, JQ698365, HQ441608, MK513209, KX306385, KC516955, KM016204, KM016198, KC018624, ON501456, OM110057, KM016172, MG549751, MG549763, MG549492, MG549605, MG549764, HQ441697, HQ993617, KC018812, JQ698344, MH593941, MT185269, MG597250, KC516953, JQ698410, HQ993905, KX790987, HQ993812, HQ993796, OM110033, ON501572, ON501546, OM110061, MF573214, MF573215, KC516899, KX306444, HQ441609, JQ698407, KM016223, JQ698396, HQ993607, KC516984, ON501474, HQ993636, HQ993966, KX306451, HQ993625, HQ993763, HQ993854, ON501576, KC018903, FJ623481, KC018605, FJ623484, OM109768, HM164123, KR086420, JQ616918, HQ993961, HQ993933, HQ993830, JQ698353, MH575300, JQ698347, KC019096, FJ623476, ON501444, HQ993786, ON501522, JQ698413, HM164120, KX306426, HQ993855, KX791042, HQ993725, KX790978, KC517009, OM110059, HQ993730, OM825070, OM825063, OM825069, OM825071, OM825065, OM825068, HQ993805, FJ623485, MH575371, MH575370, HQ993915, HQ993841, HQ993576, HQ993888, OM110096, MH575317, ON501540, ON501562, ON501557, JQ480172, HQ993706, KX306383, KT370904, KP877897, KC517036, KC516947, ON501503, HQ441678, KX790968, JX123639, JQ625607, JQ625629, DQ136764, DQ136770, DQ136771, DQ136763, DQ136762, DQ136772, DQ136765, DQ136766, DQ136767, DQ136769, DQ136774, DQ136768, KC517019, KC516888, KC516917, KT022377, HQ441749, JX123619, OM109975, KX306394, KC517005, KC516986, JQ616936, KX302538, KX302537, KX302539, KX302540, MK512922, OM110051, HQ993741, HQ993889, JQ698348, KC568505, MH317477, MH317446, MH317447, HQ993792, JX123629, KX306441, KC516913, KC516967, KP877928, KT370909, HQ993803, ON501596, KX306380, HM164131, HQ441660, HQ993596, HQ993577, MK513251, HQ993744, JQ480181, JQ480184, HQ993928, HQ441626, KT022374, KT022365, HQ993630, MH317410, MH317414, MN240783, HQ993832, HQ441650, AF457084, KC516920, JN628497, HQ993840, KC516943, HQ441657, KX306437, JN630904, OM109962, KM016196, HQ993822, KM016200, MH317309, MH317342, MH317328, MK513327, HQ993672, ON501435, MZ295236, MZ295237, KM016191, AY492759, HQ993939, HQ441599, MH575312, HQ993609, HQ441628, HQ441629, KC517058, OM109818, HQ993698, KJ185257, KJ185260, MF573219, MF573218, OM110065, HQ441601, HM164128, MH317394, KC517024, KC516918, JQ698387, MK513239, HQ993974, HQ993720, HQ993785, AM933291, HQ993655, KX306454, HQ993586, JN628477, JN132362, HQ993906, KX306417, JQ698363, JQ698359, HQ441593, KC841772, HQ993907, KJ906992, JN628485, FR666612, HQ993884, OM110098, KX306448, OM110077, MH317314, OM110079, MH317397, KR003405, MH317468, HQ993829, MH317393, MF594804, MF594803, ON501499, KX790984, MH317402, ON501507, KT022376, KT022384, MH575350, HQ993602, ON501434, ON501476, JN628501, JQ698352, MH317341, MH575335, MH575336, KT022382, JQ616923, HQ441638, JN628530, HQ993756, JN628522, HQ993930, KT022381, HQ993727, KC517049, MK513376, KX790974, KT022372, KP877912, HQ441658, JQ625631, JX123677, MH317489, JQ698415, KC568524, HQ441656, KC516879, HM164118, HQ993865, ON501492, KC018642, KC019072, KC018679, KC018842, KC517007, HQ993667, HQ993775, JQ698411, MH575339, HQ441727, KM016209, KM016190, KM016216, KM016187, KY364297, ON501473, KC517034, OM110114, HQ993908, JX123588, KX306414, JN628464, JQ698430, KC568511, HQ441616, JN630906, HQ993708, OM109820, OM110055, OM110109, MH317505, OM110117, HQ441739, OM109852, HQ441723, HQ993958, MH317346, KP877934, MH317302, FJ623477, ON501523, KX791023, KC516950, KP877906, HQ441643, HQ441699, KT022367, KX791006, KT370896, OM109976, HQ993866, ON501615, ON501538, HQ993723, KX306468, KC517048, MK922805, HQ441698, MK513274, OM109981, HQ993821, OM109982, HQ993588, HQ993968, OM110035, HQ441644, MH575331, MK513398, KC019043, HQ441696, OM824614, OM824620, OM824610, ON959679, ON501442, FJ623487, KC019003, KC018922, OM109886, MK513153, OM109863, KC516975, KX306461, KC568501, MK513002, HQ441675, JN628468, OM109825, MK192621, MK192618, KC018538, KF716469, OM109786, KC018544, KC018750, OM110137, OM110278, MH317365, OM110092, HQ441645, HQ441639, HQ441640, MH317513, HQ993633, KT833391, KT833476, HQ993867, MH317431, KC516961, KT370875, KP877917, JQ698357, MH575306, KC516890, OM109816, KM016182, OM110088, MH317361, KY364291, KC568521, JQ698397, HQ993897, OM110007, KX306453, JQ698428, JQ698402, KX306376, KC517033, MH575359, MH575358, HQ993661, KY364293, JX123609, ON501578, KC018573, KP877929, KP877927, OM109964, MH317517, JX123607, JN628482, ON501551, JN132300, MK513370, MT185157, MT185297, MT185298, KM016176, JX123604, MK513387, ON501521, KC516929, MK513290, KX306469, HQ993589, AF457052, ON501525, KC018852, KC019099, MT185161, AF457080, KM016210, KC516971, HQ993904, HQ993826, KC516926, HQ441625, JQ616937, JQ625651, JX123651, AF457068, KC516887, KR003383, OM109867, KX791036, HQ993621, KX791026, KC018926, FJ623479, HQ993851, MH317399, HQ993901, OM109850, OM109775, KC018868, JN132359, ON501541, KX791037, OM109838, KC568520, MF594802, MF594801, ON501517, OM109848, MH317407, MH317408, MH317485, KP877944, OM109887, OM109839, OM109873, KT022361, ON501495, HQ993827, MH317519, MH317521, KC516994, KP877893, HQ993973, MH317516, MH317518, MH317515, HQ993767, KY364308, JN628516, KX791046, KX790989, ON501486, JQ616917, KP877896, HQ441688, MH317411, MH317412, KX791040, KX791039, MH317354, ON501587, MH317508, ON501527, ON501528, MH317339, OM110040, KT370914, HM164117, FJ623488, KX791021, KT022380, KM016201, KC018897, KP877902, KT370861, OM110053, ON501560, KC018853, OM110004, MH317370, MH317369, OM110030, OM110104, KP877889, MG597249, OM109993, JX123584, JN628492, KY364315, OM109787, ON501608, MH317454, OM110070, OM110087, MK512896, MH317345, HQ993688, KY364294, OM110102, OM109881, OM110272, KC516969, OM110097, OM110100, MW443162, MW443161, MW443157, KC516948, ON501465, HQ441713, KC019052, FJ623475, ON501591, MH575354, MH575355, JQ698360, JQ698417, MH317451, KM016184, MH317435, HQ993780, KM016215, MH317473, HQ993769, MK513364, MT185164, OM110062, OM109865, OM110005, MH317332, KX306415, MK513301, HQ993606, HQ993573, ON501500, OM110110, ON501524, MH317439, KR003381, OM110003, OM110022, MH317507, HQ993839, MK513168, MH317484, MK512822, KR003398, KR003387, MH317396, JQ698429, MH317336, OM110046, OM109846, MH317371, OM110000, JX123582, JQ625640, JN628471, MH317318, MK513117, KY364292, OM109836, OM109884, OM109967, OM110111, OM110038, MK512807, MH317350, HQ993637, KC516922, KC516928, OM110121, ON501552, MH317499, JN628466, KX790995, KY364325, FJ623483, JN132273, JN132272, ON501610, JN628502, HQ993715, MH317395, MK192603, MK512952, MK513205, MH317470, OM109821, ON501443, KY364329, KY364307, JN628509, MH317312, MH317316, HQ993825, MZ295239, KM016207, KC516977, KM016171, JN628523, OM109870, JX123572, AF457076, KX791014, OM110089, KC018767, HQ993675, KX306470, ON501595, HQ441719, OM110080, OM109995, OM109810, MH317486, JN132360, OM109864, MH317379, KP877945, OM110049, MH317364, OM110084, JQ698371, MH317373, OM109984, OM110012, OM110118, ON959690, ON959685, ON959681, ON959686, OM824819, ON959687, OM824818, KC516910, MH317353, KT370869, KM016195, MH575338, HQ441706, KC018578, KC019000, HQ993816, JX123600, KT022364, KM016221, HQ993762, HQ993768, AF457063, AF457055, MG435520, EF368909, JQ625624, HQ993800, MT993033, JN628496, KC517053, KC516983, ON501536, MH317305, HQ993761, HQ993722, OM109854, OM110029, OM110039, MH317514, HQ993956, KC516931, KC516974, HQ993880, KX662385, MG435422, FJ389076, KT370883, JQ616931, HQ993703, KX306422, KP877911, HQ993845, JQ480177, HQ993635, HQ993642, KC517035, HQ441674, KP877900, KX791043, ON501545, AY492766, KC516895, JQ616924, OM110042, JQ071489, MG435660, KC516938, KX306410, KX306425, HQ993951, HQ993857, JN630908, MH317398, HQ993731, OM109875, KM016186, ON501493, MH317458, KC517010, KC516978, ON501613, KX791028, KX790973, ON501482, ON501459, AF457089, JN628483, MF573217, MF573216, HQ993953, KT022375, JN628505, HQ993874, KC516968, MH317390, MH317329, ON501451, ON501470, OM109841, HQ993666, KX306474, HQ993972, JN630907, HQ993898, KT022378, KM016212, ON501439, JN628461, JQ616925, HQ993782, HQ993664, HQ441683, KC516892, OM110081, KT370866, KP877908, JQ616930, KX306473, HQ993743, KT370862, KP877903, KP877936, KT370916, OM824997, OM824994, OM824998, KC018939, MG435976, OM109819, MT185296, JX460269, KJ906844, HQ993716, KY579607, MG435540, MF138697, JQ364539, KC513728, AY435236, EF612160, HG780710, KC841721, KC513715, JQ364526, AB285785, KJ906737, KC513717, JQ364522, AY955750, MF565650, EF612098, KC513716, KC841687, OM109880, MN179177, HQ993765, AB098333, JN652167, HQ993809, JQ698422, HQ993941, AY435320, KU498341, OM109977, JN628495, EF612111, AY955743, AY435331, ON501460, JQ616914, OM109815, KC517050, KC517013, KY579528, KC841708, JQ480169, HQ993859, KX790972, HG780616, KP877955, KT370895, MK192619, ON501565, AF457081, JN132361, JN630900, ON501569, KC517012, MH705151, ON501534, OM110083, KX790966, HQ441607, KX306449, KP877910, KC841691, OM110086, MH317299, HQ993931, HQ993925, JQ698362, KX790971, KX790988, JQ698351, HQ441701, JQ698354, HQ993965, HQ441744, KC568484, JX123596, ON501526, KR003400, KT370890, KX791047, KX791008, HQ993811, HQ993860, EU110085, GQ462417, AY611715, EF612002, AY428672, KF530931, MH366981, MH366916, EU251825, EU251815, KX306403, AY322190, KX302477, KX302513, KX302475, KX302476, JN628491, KC516945, AY322193, KT022373, ON501585, HQ993651, HQ993758, HQ993749, HQ993885, HQ441737, KC516896, OM109835, JQ071459, HQ993920, KC568513, JQ698374, KC568528, JQ698425, HQ993877, KC516912, JQ698406, HQ441687, JQ698343, HQ993581, KC516982, MH317326, KC517025, JN628470, JX460433, HQ993620, HQ993876, KX791035, ON501543, HQ441703, JQ698345, MK513232, KC568506, JQ698355, HQ441647, KT370872, JX123606, JQ625633, HQ441627, HQ993740, JQ480176, HQ441661, KP681726, KC568530, MH575361, MH575362, JQ698427, HQ993721, HQ441659, JN628512, EU251808, AF457065, JQ698412, KC517000, OM109849, KX791017, MH575298, HQ441702, ON501454, OM110091, JN628503, MH317308, MK513140, HQ993764, JQ698369, KC018995, KC517030, KX306389, HQ993738, KM016203, OM110058, HQ441704, KX790964, AY492777, KC516996, MH317434, OM109771, OM110050, JQ698392, HQ441633, KX791024, KX791018, MH317461, OM109826, MH317497, KT370886, JN132274, OM110041, HQ441667, JQ698356, KX306388, OM110090, ON501602, MH317386, MH317333, MH317491, DQ136749, DQ136752, DQ136754, DQ136742, DQ136761, DQ136746, DQ136751, DQ136748, DQ136750, DQ136744, DQ136760, DQ136756, DQ136759, DQ136758, DQ136757, DQ136633, DQ136641, DQ136635, DQ136640, DQ136643, DQ136639, DQ136634, DQ136644, DQ136642, DQ136636, DQ136638, DQ136637, AY435302, EF612069, KC517052, OM110060, AY444182, GQ400555, AF447846, AB485632, AY435398, AF457079, KC516941, AF410230, DQ079848, AY435364, EU110088, HQ993843, HQ993598, EF612135, AF457083, KC516985, KX306445, KC018944, KC516992, HQ993643, MK192551, MK192555, MK192560, HQ993631, HQ993887, MN650463, HQ993719, KJ906649, HQ993676, DQ136666, DQ136659, DQ136667, DQ136665, DQ136651, DQ136662, DQ136653, DQ136650, DQ136652, DQ136656, DQ136649, DQ136654, DQ136660, DQ136657, DQ136661, KX306421, HQ993912, KC516956, KC018919, JN628486, KC568525, MH317524, KC516880, HQ441689, KC019002, OM109830, JQ625622, JQ625604, KC018959, KM016202, HQ993856, OM110103, KC568486, KX306408, KT022363, HQ993610, HQ441637, MH575356, MH575357, HQ993946, KP877935, KT370915, MK513220, KC517059, HQ441615, KX306396, HQ441618, KX306398, HQ441670, KC516962, KC516878, MK513182, HQ993940, KX790967, HQ993754, KC516952, ON501491, HQ993583, JQ698368, KC516891, ON501449, HQ993654, JN628478, HQ441642, KC517055, JQ698416, KX306464, KX306467, JN628506, KC517004, KT022369, HQ993823, KC568523, JQ698409, HQ993783, HQ993970, KC018567, KR003397, KC516907, JN628465, KC517046, FJ623480, KC018634, OM109834, KC018913, MK513144, KC019083, KC018961, HQ441652, KC018952, KC018905, OM109827, OM109860, OM109828, OM109879, MK192605, MT416664, OM110274, MH317409, MT993163, KC568510, JQ698366, JN628498, HQ993603, HQ993892, KC516999, HQ441745, HQ441614, OM109989, KC018536, KC018921, KC018934, KC018621, EU380762, MG435674, KX306442, KM016173, MH317467, ON501472, JQ698401, OM109832, OM109833, OM109831, JQ698423, KC568518, JQ698386, HQ993585, MH317372, JN628519, JN628467, KC516911, HQ441676, MH317389, KY364312, JQ625658, MH575308, HQ441594, MH575307, KC568507, HQ993950, ON501511, KC517027, KC517045, ON501483, MK513243, JQ698424, KX306411, KC516921, KX306390, HQ993645, HQ993578, HQ441662, HQ441619, KC568514, HQ993680, OM109829, JN630896, HQ993616, MK513260, OM110054, MK513373, HQ993781, JQ698370, JN628489, KP877905, HQ993847, HQ993724, JQ698418, ON501488, HM164126, KR086419, HM164121, OM109808, ON959703, ON501481, ON501607, MH317481, OM110112, AF539405, HQ441743, KC516919, OM110094, DQ136795, KX790998, HQ441692, HQ441686, KC018948, HQ993634, JN628528, HQ993713, HQ993745, MH575303, MH575302, JQ698350, JQ480180, HQ993890, HQ441600, MK513082, HQ441736, HQ441684, KC568529, HQ441605, HQ993747, HQ993618, HQ993902, KX791034, HQ441729, ON501549, KC516904, HQ441728, HQ441592, OM109851, MH575314, OM109988, KC516873, KC516893, MH575348, KX791041, HQ441726, KC517028, ON501590, HQ441711, KC517040, KP877954, MZ295242, MH317480, KM016206, KC018977, MH317452, MH317478, KC568493, MH317506, ON501583, KP877959, MH317471, KC568495, OM109695, MK512874, KX306395, KX306432, MK513295, HQ441680, HQ441617, KP877888, MH575373, MH575372, ON501532, ON501612, KT022360, MW443149, MW443147, MW443148, MW443155, KX790969, HQ993960, JQ480188, OM109877, MH317464, MH317416, MK513048, MH317349, KY364322, OM110108, KY364341, KX791004, JX123577, KX791049, MH317300, MH317303, MK512916, KX306420, KM016188, MK512814, OM110273, HQ993964, ON501614, JN628513, ON501550, ON501479, ON501520, KX790994, MH317384, MH317504, KX306455, MH317377, MH317415, KC517044, JX123608, OM110036, OM110048, OM110032, HQ993683, KY364323, KR003386, OM110063, OM109994, MH575315, OM110282, OM109817, HQ993944, KX306393, OM109979, ON501539, ON501553, OM110076, KC018991, MH317320, MH317381, OM110043, MH317462, MH317430, MH575344, MH575345, OM109837, JN132302, MK512837, KM016183, OM110099, OM110085, OM109769, OM110074, ON501446, MK513263, HQ441747, MH317511, ON501502, MH317453, OM110075, KY364335, OM109985, MH317472, MG597252, OM109957, OM109960, OM109855, OM110056, OM109845, OM109770, OM110023, OM110052, OM109866, OM109813, ON501531, JQ480186, OM110120, OM109882, OM109973, MK513113, MH317476, MN240781, OM109876, MH317479, MN240789, ON501455, MK513164, MK512781, ON501561, OM109954, KX306416, JN628532, MK513029, OM109969, KX790980, OM110047, MG549585, OM109987, OM109974, KR003380, OM825091, OM825090, OM825093, OM825086, OM825085, OM825088, ON501598, MK513103, KC018645, KX790985, JN132275, MH317425, ON501457, KR003384, KR003392, MH317522, OM109968, MH317380, OM109872, OM109952, MH317465, OM110101, OM110031, OM109812, KM016217, OM109992, JN628529, OM109878, KC019067, MH317404, KC568509, MH317463, KP877946, OM110001, OM110095, OM110066, OM110069, MH317428, OM109888, MH317297, OM110131, KR003389, OM110105, OM110028, MH317403, MH317356, JN628510, OM109950, OM109953, ON501580, OM110020, OM109871, OM109811, OM109990, OM109856, OM109966, OM110044, OM109965, OM109983, KM016174, OM110071, OM109847, OM109999, OM109956, OM109972, OM109842, OM109991, OM110008, OM109955, OM109869, OM109874, OM109978, OM110064, OM109959, OM109971, OM110119, OM109963, OM110045, MN240773, OM110034, OM109980, OM109844, OM109868, OM109961, OM110122, OM109970, OM110024, OM110037, OM110116, ON501573, OM109951, MN240774, OM110093, OM110002, OM110115

**HIV subtype A1C background sequences (*n* = 157)**

KY580540, KY580520, KY580535, KY580530, KY580521, KY580539, MN791316, KY580537, KY580528, MN791313, KY580534, MN791314, KY580517, KY580531, KY580536, KC516942, AY435268, AF457087, AJ583735, KT022408, HQ993772, EF369057, HQ993629, KX306418, MH575353, MH575352, KR860886, KR860682, EF026185, KT860400, GQ433852, KM050118, KR861251, AY829296, KM050087, KR861235, GQ400646, KC018848, HM120085, KM050634, MN791514, KM050238, HM119988, MG436342, MG436301, MG436322, MG436272, KC019113, HM119631, EF381904, GQ400394, HQ993717, MT084919, KX306466, MN179226, JX300382, KC018650, ON501594, HQ993903, GQ400964, MT185159, HQ993728, MH317405, KX790990, OM110203, KX790975, JN628479, HQ993674, OM110132, OM110128, KC517041, HQ993622, KC516958, KX302525, KX302524, KX302488, ON501437, ON501480, ON501466, OM110130, OM110133, HQ993579, ON959700, ON959691, ON959697, KF716469, KF716471, KT022413, FJ623489, AF539404, AF539406, KU749431, KT022391, AF457064, KM016192, KC516993, KR003402, KC516959, KC517032, KC517060, ON501478, ON501566, HQ993710, ON501469, ON959720, ON959716, ON959718, ON959715, KC568500, HQ441694, KC568492, JQ625630, JQ625646, HQ993861, HQ993853, OM110142, OM110068, JQ625615, JQ625645, JN628469, HQ993899, MH575363, MH575364, KT022395, KM016222, KC019016, MK192549, JQ698373, KC568512, HQ441668, AY492769, AF457061, HQ441649, HQ993705, HQ993582, KM016218, KC018597, ON501603, ON501533, ON501462, KP877904, KT022393, MT185160, ON501506, KX306440, HQ993711, ON501599, ON501584, JN628481, MK513393, JX123678, OM110134, JN630899, KP877898, ON501468, OM110107, OM110187

**HIV subtype A1D background sequences (*n* = 476)**

MN791766, MN791792, MN791785, MN791791, MN791779, MN791790, MN791786, MN791777, MN791780, MN791781, MN791798, MN791789, AY322184, JN132304, GQ400857, KY579438, KX692024, JX460024, KJ907060, AJ583720, KC841661, AF457063, AF004885, KU498393, KU498414, KX662375, FJ647149, MH367009, KC516908, KU749431, KY579435, AY492781, AY492773, MG435678, AM706405, FJ623478, HQ441635, KY579571, KT022399, MG436626, HQ993696, KP681726, AF539404, AF539406, AF457078, KJ185257, HQ993848, KT022387, KT022413, KT022404, KC516879, OM824988, KF716468, KF716469, KF716471, JQ625630, HQ441694, KC568500, HQ441725, AY322191, KM016192, HQ993838, KP877910, KX302524, KX302525, KX302488, KC517041, HQ993622, KC516958, ON959695, ON959691, ON959697, AF457073, HQ993967, KT022391, KC516946, AF457064, KT022390, JQ698367, FJ623489, HQ993853, HQ993861, MK192576, FJ623490, KT022394, MH317459, KR138542, HM164127, MN816754, KX302446, HQ993760, AY492752, HQ993574, HQ441672, KY579609, JN630902, KM016211, KC517018, JN628480, KX791033, KC516988, JQ698395, ON501447, HQ441731, KC568496, JN132276, ON501498, MK192546, MK192578, MK192548, HQ993681, MH317362, MH317363, HQ441690, KT370884, KT022389, KX302426, KX302471, KX302511, KX302470, KX306386, ON501436, MH317383, KC516925, KT022412, KF531405, KF531401, KF531411, KF531404, KF531417, KF531403, KF531406, KF531400, KJ907170, HQ993818, KC516915, KM016181, AY492782, HQ993776, AY492763, HQ993863, KJ907074, KX791011, HQ441724, HQ441717, KT022397, ON501568, ON501487, KC517020, HQ993893, OM110150, HQ993657, HQ993701, KC517003, KC517021, GQ477446, EF158040, AB703615, GQ477445, EF158042, KC018856, HQ441734, KX791010, HM215250, KC516903, MH705161, JN628474, KC516959, KC517060, KC517032, ON501518, KM016178, HQ993784, JN628499, HQ441712, JN628527, AF457058, KT370900, ON501478, KR003402, KC516993, KX791045, KX791044, KY580532, KY580535, KY580520, KY580521, KY580530, MN791316, KY580528, KY580539, KY580534, MN791313, KY580531, MN791314, KY580537, KY580517, KY580536, KY580540, MT185159, ON501594, AF457087, HQ993717, HQ993728, HQ993772, OM110203, KX790990, KX306418, MH317405, KX306466, JN628479, KX790975, HQ993629, ON501452, KX791016, KX791020, KX790991, ON501466, ON501480, HQ993674, ON501566, HQ993711, KT022395, KM016222, KC516942, KT022408, JQ698378, KT022392, KC517026, OM109998, ON501563, JQ616941, EU110090, ON501513, KX791019, OM110072, KC516981, AY492755, AY492757, JQ616915, HQ993842, ON501519, KX306443, JN132357, HQ993918, HQ993881, KX306446, JQ698376, KM016213, HM164124, HM164113, HQ993600, HQ993914, HQ441596, KX306447, MH317315, KX306428, KX306456, KX306471, JQ698381, ON501516, OM110161, KC517008, OM110158, OM110163, OM110165, HQ441602, KT022396, FJ623491, HQ993787, KR003393, ON959720, ON959718, ON959716, ON959715, JQ625598, OM110211, MN694999, MG436336, KT370905, KP877925, MH317501, MH317502, ON501567, KR138541, HM164112, KX306450, KT370885, JQ698389, KT022417, ON501458, MH317500, ON501477, KC516906, KC516902, MN791449, MN791444, OM110162, KT022405, AY492762, OM825117, OM825122, OM825123, OM825120, OM825118, OM825121, OM825119, AY492776, KT022388, KT022409, KT022385, KC517002, HQ441598, HQ993714, KP174771, HQ993587, KC516905, KC516960, KT022410, KX306427, KX306458, AF457059, KT022416, KT022411, KT370878, KT370898, ON501588, OM110267, KY364290, ON501577, OM110167, JQ616929, ON501558, OM110141, OM110156, AF457082, KX306462, JN628462, ON501496, KC018553, MH317437, MN240782, OM110139, KX791030, JN630898, KY364316, KX306431, MH575353, MH575352, HQ993903, AF457061, AY492769, JQ698373, KC568512, HQ441668, MK192549, KC019016, KC018597, HQ441649, HQ993582, HQ993705, KM016218, ON501603, ON501533, JQ625645, JQ625615, OM110068, JN628469, HQ993899, ON501469, HQ993710, ON501462, KP877938, KT022393, MH575364, MH575363, HQ993718, HQ993836, ON501506, ON501437, OM110128, OM110132, OM110130, HQ993579, MK513393, JN628515, JN628514, HM164116, OM110159, MH317355, KP877915, KT370873, KR003385, KT370917, KP877898, ON501584, KX306440, MK192613, MK192588, OM110142, OM109997, OM110140, MK512933, ON501599, OM110134, OM110164, OM110160, KM016220, MH317421, OM110209, MH317337, KC516901, MK513149, OM110210, ON501555, KP877931, KT370911, HQ993659, ON501581, ON501497, JN628517, KC516898, HQ993922, KY364295, KP877947, OM110153, OM110067, KM016189, MN791641, MN791635, MN791646, MN791653, MN791654, MN791667, MN791643, MN791633, MN791648, MN791652, MN791660, MN791637, MN791662, MN791656, MN791645, MN791666, MN791664, MN791663, MN791661, MN791655, MN791634, MN791658, MH317359, JN630899, MT185160, JN628481, OM110133, JX123678, ON501537, ON501468, OM110078, OM110152, MH317483, MH317401, MH317455, KR003391, JN132358, OM110157, OM110154, JN628525, OM110124, OM110006, OM110151, OM110143, OM110155, OM110107, OM110244, OM110187

**HIV subtype B background sequences (*n* = 28)**

KT370913, MT908058, MT908073, MH470811, MH471306, HM043651, MH472100, MH472216, KJ770580, KJ770967, KJ771283, KJ771114, KJ771268, HM043648, KJ770367, MH471398, MH471402, MH471514, KJ771402, GQ399180, EU248339, KJ771085, KJ771370, KC018507, JQ650662, MH472416, OP441576, MH472251

**HIV subtype C background sequences (*n* = 256)**

MN791503, MN791531, MN791528, MN791526, MN791518, MN791514, MN791525, MN791524, MN791530, MN791533, MN791529, MN791523, MN791527, MN791506, MN791500, MN791508, MN791495, MN791521, MN791496, MN791502, HQ993831, HQ993833, KC018599, JQ625656, KF736544, KT183072, KT183068, GQ427128, AB485645, AB485646, AB485647, KM050646, KM050645, MH710098, KM049902, HE588162, GQ433812, KR861000, KX791468, KY099003, MF278415, KC423638, MG435847, KM050514, KU498864, JN314975, DQ826615, MH757194, KT860358, HQ441610, HQ441611, MT194492, MT194484, ON501554, KC516875, KC516987, AY492764, KC516954, HQ993952, AF457054, JN628493, KC517051, MH355426, MH353472, KX791490, KU498867, HQ993969, HQ993692, MH710331, HQ993926, KC516889, HQ993789, KC517038, HQ993886, HQ993580, AY756851, OM110198, HQ993813, JQ480174, HQ993684, HQ993699, ON501509, KC516939, MK512946, HQ993837, HM164129, HQ993597, KC517043, KX306409, HQ993623, MH575320, MH575319, KJ906795, KF530993, KF530994, AM260292, HE588161, KU821011, KU821008, KU821013, KM050441, AM260278, HG780773, EU248453, EU248561, HG780712, AY563170, HG780662, KP681722, HG780798, MH366905, HQ441632, KM049950, HQ993962, MH366868, OM110195, MZ295233, MZ295234, HQ993938, HQ993937, AY945738, KC018824, HG780716, MH366948, HG780656, MG436322, MF138737, KU253010, OM110192, AJ583722, KC517029, MF594799, MF594800, HQ993678, HQ993943, JQ480185, HQ993963, HQ441720, HQ993632, KP877942, KT370874, KC517037, MK513351, HQ441732, KM016194, MK512795, KC018732, KC517042, HM119781, OM110197, HQ441624, KT022362, KX306429, HQ441612, HQ993806, HQ993858, HQ993702, HQ993927, OM110177, HQ441682, HQ993690, OM110189, KX790977, KT370865, KP877939, KX791001, ON501600, JQ698341, HQ993653, HQ993615, JN630894, MK512798, HQ441669, MH575332, HQ993750, HQ993949, JQ616928, KX790965, OM110196, HQ441641, HQ993729, HQ993734, MT185158, KC517017, HQ993891, KM016208, JN628518, HQ993849, KT022371, HQ993909, JN628500, HQ844613, HQ993611, HQ441648, OM110178, KX791022, KY364340, MH317429, KT022366, KC018664, HQ993819, OM110206, KP877957, MH317418, KC018516, OM110190, MK513268, OM110182, ON501438, JN628507, JQ698405, KP877892, JQ698393, ON501592, MH317392, MK192592, MK192623, KY364301, MH317494, MH317424, OM110202, OM110205, MH317426, OM110204, KY364289, JQ616927, MH317520, OM110200, MK512817, MH575360, OM110185, MH317466, MH317492, MH317375, MH317374, HQ441604, MH575328, MH575327, KC516970, MH317417, MZ295235, OM110186, MZ295241, OM110175, JN132305, OM110174, MK512960, MH317321, ON501489, MH317456, MH317523, KX306377, OM110183, OM110191, OM110184, OM110176

**HIV subtype D background sequences (*n* = 443)**

OM825010, OM825011, OM825003, MN791466, MN791491, MN791488, MN791458, MN791459, MN791468, MN791484, MN791475, MN791490, OM110229, JQ616934, JX123637, ON501601, OM825106, OM825109, OM825107, OM825113, MK192611, KF716476, MK192595, MK192593, HQ441621, MK192564, MK192572, DQ136688, DQ136685, DQ136687, DQ136684, KJ906801, AY435258, EF612013, AY444270, AY955772, EF612172, AY435388, EF612124, FJ389149, FJ389090, AY428725, EF612161, FJ389055, AF388147, EU251720, EF612034, EU251850, MT993149, KP877951, KX306460, JQ625618, JN630897, AF457090, HQ441665, AY444269, AF388157, AY492783, HQ993594, AF410203, MW316920, HQ441666, JQ698399, KC568522, AY492779, KR003382, HQ993900, GQ399143, KC018747, ON501441, HQ993601, MW006081, AY492771, KX790993, KC516914, KX791002, JQ698358, KC568504, JQ698346, KC517001, JX123601, HQ993895, HQ993795, JQ698408, HQ441654, EF612035, JX123670, ON501505, KX306459, KX306423, AY492760, HQ993779, KC516933, KC516924, HQ993590, KC516940, KC516949, KC516972, OM110223, KC516916, MK513318, AY322189, HQ441691, JQ480182, HQ993641, ON501530, HQ993751, KX306413, KC516885, KC018789, ON501510, HQ441591, KX790992, KC516900, HQ993947, HQ993913, KX791027, MK513390, JQ698388, MT993225, HQ993810, KC516894, KT370902, KP877894, HQ993755, HQ993605, HQ993593, HQ993663, KT370912, KP877932, JQ698426, KC516876, KC568527, MH575305, HQ441716, OM110254, KC517014, HQ993929, HQ993921, KC516989, KC516936, MK513304, KX791032, HQ993852, KC517047, HQ993799, KC516883, AY492758, HQ993662, KC517056, AY492768, HQ993673, OM110280, KX790976, KX791025, KX306452, HQ993612, HQ993595, KM016199, KX790970, KX791050, KC516979, KP877909, KT370867, KR003404, KC018631, AY435307, EF612063, KX790979, AY435352, MK512852, KT370882, KP877961, KX907417, KX907430, KX907411, AF457072, KX306419, OM109723, OM109724, OK181202, EU342768, M27323, MG470653, FJ591687, K03454, DQ344020, M22639, KM057352, AM040994, AY945737, AM041035, AJ419462, HQ127582, DQ138972, GQ400336, AJ287019, AY102509, KP877956, GQ400071, KX306399, JX161341, MN178957, KC516881, MH705152, KC516966, KX791015, HQ441623, HQ441677, HQ993707, KX790997, KM016185, ON501514, KX306438, KC018954, HQ993942, KC018707, KC018828, KC018525, KP877948, OM110266, JN630905, MH317460, JQ616926, MH317490, HQ441695, JQ698372, JN628476, HQ441664, ON501579, KX791012, JN628484, HQ993948, DQ136672, DQ136670, DQ136674, DQ136676, DQ136671, DQ136679, DQ136677, DQ136673, DQ136682, DQ136669, DQ136675, DQ136680, AY492788, HQ993807, HQ441622, JX123628, KM016205, OM110257, OM110265, JQ698404, JQ698391, KX791009, KC018938, KC018530, OM110279, HQ993604, HQ993656, KM016177, KC516897, KC568478, KC568479, MH317338, OM110261, ON501589, MH317376, OM110250, MF594798, MF594797, HQ441655, JX123574, AY444267, MH317469, MK512906, KY364336, MH317334, JX123675, JX123627, JQ616935, KT370887, KC516930, HQ993753, KP877913, KT370868, KM016219, HM164119, ON501570, KC018711, MH317317, HQ993955, KT370876, KP877918, HQ993924, KC516973, HQ993869, ON501464, ON501471, ON501609, JQ625606, JX123646, AY492789, MH317475, MH317474, MH317436, MH317319, OM110262, MH317427, JX123576, KC516980, OM110252, OM110221, OM110253, KX791031, MH317360, ON501586, HQ993682, KR003388, OM110231, JN132306, MH317391, MK513057, MH317387, KM016193, KM016175, KX790982, KC516995, JQ616916, KX306475, MF403423, MH317498, JQ480187, JQ698394, KX791007, OM110227, HQ993685, JQ480178, MK513308, KX791029, OM110226, OM110225, JQ698340, JQ698382, MK192622, MH317423, KR003394, ON501604, KX306404, OM110259, OM110256, MH317406, OM110260, MK512858, KM016180, OM110263, HQ993896, MK513211, MH317306, OM109726, MH317445, HQ993739, ON501467, KX306463, KC516963, KX306465, KX791003, HQ993599, JN393302, MK513248, HQ993975, MH317496, ON501475, JN630895, OM825099, OM825098, OM825095, OM825094, OM825100, OM825102, OM825096, OM110258, ON501529, ON501574, OM110230, OM110248, MH317493, OM110241, MH317510, MH317503, MH317351, MG597253, MH317432, OM110224, KY364339, ON501450, ON501571, MH317450, OM110264, OM110247, OM110251, MH575321, OM110216, OM110222, OM110255, OM110243, OM110249, OM110242, KR003403, OM110138, MH317413, OM110228

**HIV subtype G background sequences (*n* = 49)**

AB640341, AY444285, MH705155, HQ267577, KC340465, AJ286997, FN599725, FJ577269, JN673639, FN557322, FJ577283, FN599703, FJ577351, MN163456, MN908774, MN908780, JX301013, KY579688, MH654837, MH654940, MH654858, MH654931, MK784441, MK784483, MH654826, AB485662, AB485663, AF061640, AF061641, AF447856, AY492756, HQ441714, HQ993704, HQ993709, HQ993788, HQ993824, JN630903, JQ914088, KC018675, KC516874, KC517039, MT908068, OM110269, OM110270, OM110271, OQ299155, OQ299193, OQ299324, OR508331
